# Meta‐analysis indicates that oxidative stress is both a constraint on and a cost of growth

**DOI:** 10.1002/ece3.2080

**Published:** 2016-03-21

**Authors:** Shona M. Smith, Ruedi G. Nager, David Costantini

**Affiliations:** ^1^ Institute of Biodiversity, Animal Health & Comparative MedicineUniversity of GlasgowGraham Kerr BuildingGlasgowG12 8QQUK; ^2^ Department of BiologyUniversity of AntwerpAntwerp2610Belgium

**Keywords:** Antioxidants, enzymes, growth rate, life‐history theory, oxidative damage, reactive oxygen species, trade‐offs

## Abstract

Oxidative stress (OS) as a proximate mechanism for life‐history trade‐offs is widespread in the literature. One such resource allocation trade‐off involves growth rate, and theory suggests that OS might act as both a constraint on and a cost of growth, yet studies investigating this have produced conflicting results. Here, we use meta‐analysis to investigate whether increased OS levels impact on growth (OS as a constraint on growth) and whether greater growth rates can increase OS (OS as a cost of growth). The role of OS as a constraint on growth was supported by the meta‐analysis. Greater OS, in terms of either increased damage or reduced levels of antioxidants, was associated with reduced growth although the effect depended on the experimental manipulation used. Our results also support an oxidative cost of growth, at least in terms of increased oxidative damage, although faster growth was not associated with a change in antioxidant levels. These findings that OS can act as a constraint on growth support theoretical links between OS and animal life histories and provide evidence for a growth–self‐maintenance trade‐off. Furthermore, the apparent oxidative costs of growth imply individuals cannot alter this trade‐off when faced with enhanced growth. We offer a starting platform for future research and recommend the use of oxidative damage biomarkers in nonlethal tissue to investigate the growth–OS relationship further.

## Introduction

Animals do not appear to grow at the maximum rate (Blanckenhorn 2000) which is peculiar given the potential benefits of reaching an increased size quickly, including reduced predation risk, earlier time to sexual maturity, and so increased lifetime reproductive success (Dmitriew 2011). This implies there must be constraints on rapid or accelerated growth through a resource allocation trade‐off where energetically expensive growth causes resources to be diverted away from other processes such as physiological development (Metcalfe and Monaghan 2003). If individuals are not able to completely adjust that trade‐off, it may result in costs of rapid growth in terms of reduced self‐maintenance.

Growth is an energetically demanding process that diverts resources away from self‐maintenance processes. One of these processes that can be negatively affected in faster growing individuals is the level of antioxidant protection (Alonso‐Álvarez et al. 2007). An alternative mechanism by which growth might relate to self‐maintenance processes is that the increased cellular activity needed for enhanced growth leads to increased production of reactive species (RS) as a by‐product of metabolism (Mangel and Munch 2005; Dmitriew 2011). Indeed, there are several studies linking increased growth rate with increased metabolic rate (Criscuolo et al. 2008; Careau et al. 2013; Stier et al. 2014b) and daily energy expenditure (Careau et al. 2013). Increased metabolic rate can increase RS production (Mangel and Munch 2005; Dmitriew 2011; but see Barja 2007; Fletcher et al. 2013; Stier et al. 2014b; Salin et al. 2015 for a debate) – when this occurs, antioxidants will be mobilized or upregulated in order to neutralize the RS (Yu 1994). If there is an imbalance between the levels of RS and antioxidant protection, this can result in oxidative damage to tissues (Yu 1994) and a state of oxidative stress (OS). It has been suggested that OS might play a key role as a constraint on, and cost of, growth (von Schantz et al. 1999; Monaghan et al. 2009; Costantini et al. 2010; Costantini 2014).

Yet inconsistent patterns have been reported for the relationship of growth with OS when considering within‐species patterns in both correlational and experimental studies investigating the cost of growth. Faster growing individuals have been linked to both raised (Leggatt et al. 2007; Salomons 2009) and reduced (Kilgas et al. 2010; Almroth et al. 2012) antioxidant levels, while other studies have found no effect (Rosa et al. 2008; Larcombe et al. 2010; Geiger et al. 2011). Moreover, enhanced growth rates have been associated with increased oxidative damage within species that cover a number of different taxa (Nussey et al. 2009; Almroth et al. 2012; Stier et al. 2014b) but not always (Rosa et al. 2008). This confounds our understanding of the growth–OS relationship.

The apparent discrepancies between studies investigating the growth–OS relationship could be the result of different studies measuring different components of the redox system. Antioxidants have varying specificity for the vast array of RS that exist (Halliwell and Gutteridge 2007), so different antioxidants will be mobilized or upregulated depending on the RS that has been produced. Moreover, utilization of such antioxidants could result in a decrease, rather than increase, in antioxidant levels. However, either of these responses may be sufficient to neutralize the increased RS levels. If RS are not neutralized, there are numerous oxidation products that can result (Dotan et al. 2004) but many studies do not measure more than one damage biomarker. The variability in the redox response emphasizes the importance of including a number of different biomarkers of both oxidative damage and antioxidant defense when assessing whether the changes in RS and antioxidant levels impact on the organism.

In correlational studies in which phenotypic flexibility in growth has been associated with OS, it is unclear whether the relationship is causal. Experimental alteration of growth rates and comparison of OS between manipulated and unmanipulated individuals within the same species could clarify causality. For instance, reducing brood size (Salomons 2009) and increasing lipid/protein composition of the diet (Costantini 2010) have both led to increased growth rates and higher oxidative damage. The induction of compensatory growth (Metcalfe and Monaghan 2001; Dmitriew 2011) is sometimes linked to increased oxidative damage (Tarry‐Adkins et al. 2008; Hall et al. 2010) but not always (Savary‐Auzeloux et al. 2008; Noguera et al. 2011).

Additionally, the growth–OS relationship might depend on the developmental stage of the organism; for instance, at certain points in development, an individual could be more vulnerable to OS. This is particularly notable at birth/hatching due to changes in the partial oxygen pressure and metabolic rate (Surai 2002; Davis and Auten 2010). As the enzymatic antioxidant system takes time to become fully mature, there may be a greater reliance upon nonenzymatic antioxidants at earlier stages of development and mothers may compensate by increasing deposition of antioxidants into prenatal stages (Surai 2002).

The aim of this study was therefore to use meta‐analytic techniques to review evidence for the relationship between growth rate and OS within species, while taking into account some of the confounding factors, such as what biomarkers were measured, in what tissue, and at what developmental stage. A diverse range of eight taxonomic classes were considered. Two hypotheses were tested: the first was whether OS constrains growth as we might expect whether a resource allocation trade‐off is present between the two systems. In this case, increased OS levels (i.e., greater damage and/or reduced antioxidant levels) would lead to a reduction in growth rate. To test this, the first meta‐analysis (hereafter termed the constraint meta‐analysis, constraint‐MA) included studies where growth could be compared between groups that had been experimentally manipulated to differ in OS levels. This allowed us to investigate whether lower antioxidant levels might lead to a greater investment in antioxidant protection at the expense of growth, as well as whether greater oxidative damage to tissues could limit growth. If a trade‐off between growth and self‐maintenance does exist, then one might expect there also to be costs of growth, if individuals cannot adjust the trade‐off in the face of accelerated growth. Therefore, the second hypothesis to be tested was that OS is a cost of growth, where we would expect that increased growth rates would lead to greater OS levels (i.e., greater damage and/or reduced antioxidant levels). Therefore, the second meta‐analysis (henceforth referred to as the cost meta‐analysis, cost‐MA) included studies that had experimentally manipulated growth rate between individuals, which could then be compared in terms of OS to determine whether these within‐species growth differences impacted on OS. By including a vast array of biomarkers of OS over numerous species covering eight taxonomic classes, our meta‐analyses provide an in‐depth investigation of the complex interplay between growth and OS.

## Materials and Methods

### Data collection

We focused on studies that manipulated either aspects of OS or growth within species. A systematic literature search was carried out in Web of Knowledge using combinations of the keywords “growth,” “growth rate,” and “compensatory growth” with “oxidative stress,” “antioxidant,” and “oxidative damage”. In addition, a more detailed search with the same keywords but also including “supplementation,” “oxidized lipid,” and “oxidized fat” was carried out to obtain studies that had manipulated OS through dietary changes, for instance antioxidant supplementation or inclusion of oxidized fats in the diet. Singular rather than plural words were used where appropriate, and the following truncated words were used: supplement* (thus incorporating supplement, supplemented, and supplementation) and oxid* (to include oxidized and oxidative). The last search was conducted on 7 August 2014, and citations of key papers were also searched. This resulted in the screening of approximately 2410 papers, and relevant studies were included (Appendix S1). If possible for each included study, multiple effect sizes were extracted if separate measures of various antioxidant levels and damage biomarkers were provided. For testing the first hypothesis, whether OS constrains growth (constraint‐MA), there were 184 effect sizes in growth between groups where OS was manipulated from 61 studies. To investigate whether OS is a cost of growth (cost‐MA), there were 120 effect sizes in OS between groups where growth was manipulated from 28 studies. Further details of the selection procedure for inclusion of studies into the meta‐analyses, and standardization of growth measurements across studies and tests that revealed the absence of any publication bias are provided in Appendix S1 and Figure S1.

### Effect size calculation

The *compute.es* package (Del Re 2013) in R (R Core Team 2013) was used to calculate the standardized effect size Hedges' *g* from test statistics (e.g., *t* values or *F* ratios) and sample sizes that were reported in papers; this package applies appropriate formulae described in Cooper et al. (2009). To calculate effect sizes, the standardized mean difference (Hedges' *d*) was first calculated – this accounts for the use of different units (i.e., different redox biomarkers) between studies by dividing the raw difference by the within‐group standard deviation. For Hedges' *d,* the type I and II error rates can increase if the number of studies is very low (<15) but the precision of the estimate increases with increasing number of studies (unlike other effect size measures; e.g., log response ratio) (Lajeunesse and Forbes 2003). Thus, given the large sample size of the current meta‐analyses, Hedges' *d* was deemed an appropriate effect size estimate.

With small within‐study sample sizes, Hedges' *d* can be over‐estimated, so to correct for this it was converted to Hedges' *g* by multiplying by a correction factor calculated from the degrees of freedom (Cooper et al. 2009; Del Re 2013). Where appropriate test statistics were not reported, means, standard errors, and sample sizes were extracted from tables or figures using ImageJ (Abràmoff et al. 2004), which could then be entered into *compute.es*. If mixed models had been carried out in the original study and there was access to the model output, *r* was calculated from equation 24 in Nakagawa and Cuthill (2007) and then converted to Hedges' *g* as described above. Where appropriate, the number of families contributing to the dataset was used as the total sample size rather than the number of offspring, to account for nonindependence of siblings sharing the same rearing environment.

### Moderators included and categorization

As the relationship between growth rate and OS can be influenced by various factors, several explanatory variables (termed moderators in meta‐analysis) were considered to be included in the analyses. The nature of the experimental manipulation might be influential, so is an essential moderator. For constraint‐MA, three types of experimental manipulation were considered (Table 1A). For supplementation with both antioxidants and natural compounds, we expected an improvement in the antioxidant status of supplemented individuals. Therefore, unsupplemented individuals would suffer higher levels of OS and this would lead to a reduction in growth. On exposure to stressors (i.e., environmental challenges that increased OS), exposed individuals were expected to reduce their growth. For cost‐MA, we included three different types of experimental manipulation and four correlational studies (Table 1B). Regardless of treatment, we expected a greater level of OS (so increased damage and/or reduced antioxidants) in the faster growing groups.

**Table 1:** Summary of the experimental manipulations for constraint‐MA (A) and cost‐MA (B). Note that some studies provided data for more than one experimental manipulation

Experimental manipulation	Details	Sample size: studies (effect sizes)
(A)		
Supplementation with antioxidants	For example, carotenoids, vitamins, synthetic compounds that led to a reduction in OS (i.e., decreased damage and/or greater antioxidant levels), compared with the unsupplemented group	24 (63)
Supplementation with natural compounds	Compounds with potential antioxidant properties (e.g., prebiotics, probiotics, herbs, plant extracts) that led to a reduction in OS (i.e., decreased damage and/or greater antioxidant levels), compared with the unsupplemented group	23 (63)
Exposure to stressors	Environmental stressors that induced OS (i.e., increased damage and/or reduced antioxidants), for example, inclusion of oxidized lipids in the diet, exposure to hypoxia, high stocking density, heat stress, toxins	21 (58)
(B)		
None^a^	Correlational studies in which the growth difference between groups was natural and statistically significant	4 (18)
Compensatory growth	Food restriction followed by a period of ad libitum food, leading to compensatory growth in the experimental group	7 (34)
Brood manipulation	Altering the number of chicks or hatching synchrony within a brood in avian studies. This led to increased growth in reduced broods compared with controls. Enlarged broods had decreased growth rates compared with controls, as did chicks that hatched asynchronously compared with synchronously. For one study, compensatory growth occurred later in life after an initial growth decrease of enlarged broods	4 (8)
Dietary changes	Changes to protein and lipid composition of the diet. This included diets of differing quality with greater growth rates in high‐quality diet groups, as well as comparisons of different types of dietary proteins (e.g., fish meal, maggot meal, or soybean meal) and lipids (e.g., cod liver oil or vegetable oil) that had different effects on growth	13 (60)

a None included comparisons between younger and older individuals with different growth rates, initially small late‐hatched and larger early‐hatched individuals and between individuals living at different elevations.

Secondly, the growth–OS relationship is likely to depend on which biomarker is considered, because the antioxidants responding to, as well as the damage molecules produced from, OS can vary greatly. Therefore, biomarker type was included and categorized into (1) damage biomarkers that included markers of protein (e.g., protein carbonyls, PCs), DNA (e.g., 8‐oxo‐dG), and lipid (e.g., malondialdehyde, MDA) damage; (2) nonenzymatic antioxidants (e.g., thiols, carotenoids, and measures of total antioxidant capacity); and (3) antioxidant enzymes (e.g., catalase, glutathione peroxidase, and superoxide dismutase). A list of all the specific biomarkers is given in Tables S1 and S2.

The developmental stage of an organism is likely to have consequences for the growth–OS relationship because at certain developmental stages animals may become more susceptible to OS. The dataset spanned eight taxonomic classes – Actinopterygii, Amphibia, Aves, Gastropoda, Holothuroidea, Malacostraca, Mammalia, and Reptilia. Therefore, developmental stage was standardized by categorization into: (1) early juveniles (larvae of fish/insects/Malacostraca, tadpoles, nestling birds, mammals not yet weaned); (2) older juveniles (juvenile fish, fledged birds not yet of reproductive age, weaned mammals not yet of reproductive age, postlarval Malacostraca); (3) adults (i.e., of reproductive age). In constraint‐MA, there was a large sample imbalance between groups, with only two studies being from adults. Therefore, the analysis was repeated excluding the two studies on adults so that older juveniles and early juveniles could be compared – no significant difference was found so developmental stage was not included as a moderator in constraint‐MA.

Another moderator that was considered was sampling method, which was categorized into nonlethal (e.g., of blood, urine) and lethal (e.g., of liver, muscle), in order to determine whether the same effects could be obtained with nonlethal sampling. The sampling method will also affect the tissue type available for analysis, which has been suggested to lead to variations in the growth–OS relationship (Brown‐Borg and Rakoczy 2003; Leggatt et al. 2007). For a full list of studies included in both meta‐analyses and a breakdown of the categorization of specific biomarkers and tissues see Tables S1 and S2.

### Meta‐analytic technique

Meta‐analytic multilevel mixed‐effects models were implemented using the rma.mv function in the *metafor* package (Viechtbauer 2010) in R (R Core Team 2013). The extracted Hedges' *g* values were the response variables in our statistical models. For constraint‐MA, the Hedges' *g* values denoted whether groups of the same species that had been found to differ in OS levels also had significantly different growth rates (growth is the response variable). As here we are interested in whether OS constrains growth, a positive Hedges' *g* value meant that the group with the highest level of OS (i.e., greater damage and/or reduced antioxidants as OS is associated with *higher* levels of damage but *lower* levels of antioxidants) also had the *lowest* growth rate. For cost‐MA, the Hedges' *g* values indicated whether there was a significant difference in OS between two groups of the same species that had been found to differ phenotypically in growth rate (OS is the response variable), with a positive value signifying that the group with greater growth rates also had greater OS levels (defined as increased oxidative damage and/or decreased antioxidants).

Estimates were weighted according to the inverse sampling variance to account for different sample sizes across studies. This multilevel approach allowed the inclusion of study ID (to accounting for the nonindependence of effect sizes from the same study) and taxonomic class (to partly control for phylogeny, which is difficult to do as the dataset was rather unevenly distributed across eight taxonomic classes) as random effects. Experimental manipulation, biomarker type, developmental stage (cost‐MA only), and sampling method were included as moderators. The model output included the *Q*_E_‐test for residual heterogeneity, indicating whether the unexplained variance is greater than expected by chance – if it was, then there is some variance that is not accounted for by the moderators. An omnibus test of model coefficients indicated whether there were significant differences among moderator levels; the test statistic for this is *Q*_M._ Models were simplified in a stepwise manner by the removal of nonsignificant terms, using a likelihood ratio test to compare the fit of the full and reduced model at each stage. Postmodel fitting checks were carried out, firstly by plotting the (restricted) log‐likelihood against each variance component in the model, to ensure the function peaked at the parameter estimates. A flat surface around the parameter estimate would suggest the model was over‐parameterized (Viechtbauer 2014). Additionally, model residuals were checked and met the requirements for normality and a lack of heterogeneity. Finally, as an outlier in cost‐MA was detected where the Hedge's *g* was more than double the value of the next highest point (and so might have been influencing the results), models were repeated excluding it. As it made no qualitative difference to the outcome, all presented results include the outlier.

When moderators are included in meta‐analysis, it is difficult to get an overall average effect size because this will be influenced by the distribution of studies among moderators (Viechtbauer 2007). Therefore, the models that contained moderators were used to calculate predictions for particular “sets” of moderators rather than an overall effect size (e.g., for each treatment type in constraint‐MA and each biomarker type in cost‐MA).

## Results

For investigation into whether the manipulations successfully altered OS (constraint‐MA) and growth (cost‐MA), plus a discussion of whether the manipulations produced differences between groups that were similar in magnitude to the differences that occurred under unmanipulated conditions see Appendices S1–S3, Table S3, and Figure S2.

**Figure 1:**
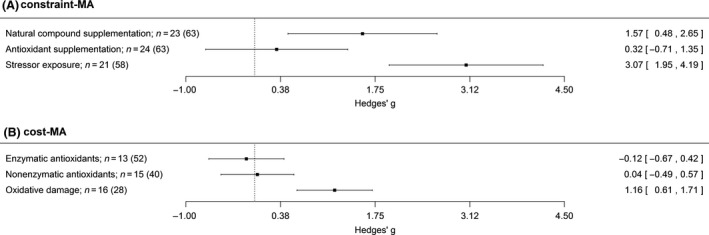
Predicted effect sizes (mean and 95% confidence interval, CI, at right) for (A) constraint‐MA (slower growth in groups with experimentally increased oxidative damage and/or reduced antioxidant defenses) and (B) cost‐MA (greater oxidative damage and/or lower antioxidant defenses in the experimental group with faster growth). A: a positive effect size indicates increased OS is associated with reduced growth. Separate effect sizes are given for each experimental manipulation, as the effect size differed significantly between these. B: a positive effect size indicates increased growth is associated with increased OS. As the effect size differed significantly between biomarkers of OS, separate effect sizes are given for each biomarker. When the CI does not include zero, the effect size is significant. “*n*” is number of studies (number of effect sizes). OS, Oxidative stress.

There was an association between raised OS levels (i.e., greater damage, such as to proteins, lipids or DNA, and/or reduced antioxidants) and reduced growth in the constraint‐MA implying OS was constraining growth. This effect was significantly different among different experimental manipulations (*Q*_M_ = 36.64, df = 2, *P* < 0.0001; Table 2A for pairwise comparisons), but not between different biomarkers (*Q*_M_ = 3.65, df = 2, *P* = 0.16) or sampling methods (*Q*_M_ = 0.41, df = 1, *P* = 0.52). Stressor exposure produced the largest effect, and this was positive and significant (Fig. 1A), suggesting exposure to environmental challenges that raised OS levels reduced growth. Supplementation with natural compound (e.g., plant extracts, probiotics, herbs that have potential antioxidant properties; not specific antioxidants) also produced a significant positive effect, but the effect for antioxidant supplementation was not significant (Fig. 1A). Therefore, supplementing with natural compounds, but not specific antioxidants, also supports the hypothesis that increased OS constrains growth; increased OS (increased damage and/or lower antioxidant levels) in the unsupplemented group was associated with reduced growth.

**Table 2:** Pairwise comparisons between effect sizes for constraint‐MA (A) and cost‐MA (B). A: comparison of the effect sizes (slower growth in groups with experimentally increased oxidative damage and/or reduced antioxidant defenses) between the different experimental manipulations; natural and antioxidant supplementation and stressor exposure. There was no significant difference among biomarkers for constraint‐MA; only experimental manipulation was left in the final model. B: comparison of effect sizes (greater oxidative damage and/or lower antioxidant defenses in the experimental group with faster growth) among the different OS biomarkers; enzymatic and nonenzymatic antioxidants and oxidative damage. There was no significant difference among manipulations for cost‐MA; only biomarker type was left in the final model. Significance did not change following adjustment of *P* values using the sequential Bonferroni method (Rice 1989). The variance explained by the random factors was 0.07 and 0.51 (taxonomic class) and 1.25 and 5.92 (study), for constraint‐MA and cost‐MA, respectively

	*z* value	*P* value	Adjusted *P* value
A. Pairwise comparisons (constraint‐MA)			
Natural–antioxidant	2.60	0.009	0.01
Natural–stressor	2.80	0.005	0.01
Antioxidant–stressor	6.03	<0.0001	0.0003
B. Pairwise comparisons (cost‐MA)			
Enzymatic–nonenzymatic	0.94	0.35	0.35
Enzymatic–damage	6.17	<0.0001	0.0002
Nonenzymatic–damage	6.45	<0.0001	0.0002

For the analysis of the effect of differences in growth on OS (effect sizes in the cost‐MA), a positive effect signified that increased growth was associated with increased OS (i.e., increased damage and/or reduced antioxidants). This effect was not significantly different between different experimental manipulations (*Q*_M_ = 0.38, df = 3, *P* = 0.95), developmental stages (*Q*_M_ = 0.35, df = 2, *P* = 0.84), or sampling methods (*Q*_M_ = 2.44, df = 1, *P* = 0.12). However, there was a significant difference among different biomarkers of OS (*Q*_M_ = 49.46, df = 2, *P* < 0.0001; Table 2B for pairwise comparisons), with markers of oxidative damage producing significantly larger effects than those of enzymatic or nonenzymatic antioxidants (Fig. 1B). Furthermore, only oxidative damage produced a significant positive effect (Fig. 1B).

The *Q*_E_‐test revealed significant levels of residual heterogeneity (constraint‐MA: *Q*_E181_ = 1108.40, *P* < 0.0001; cost‐MA: *Q*_E117_ = 553.86, *P* < 0.0001) implying that the variance not accounted for by the moderators was significantly greater than expected, and thus, it is likely that there are additional moderators not considered here that might be responsible for the residual variation.

## Discussion

In using meta‐analysis to review available data on the relationship between growth rate and OS within species, we found that experimentally altering OS levels so that OS was increased (increased damage and/or reduced antioxidants) through exposure to environmental stressors or being unsupplemented (compared with groups that were supplemented with natural compounds but not antioxidants) was associated with a reduction in growth rate, suggesting OS constrains growth. This effect was significantly influenced by the type of experimental manipulation but not biomarker type or sampling method. Furthermore, animals with phenotypically greater growth rates had higher levels of oxidative damage, although no difference in antioxidant levels. This indicates that there are costs in terms of oxidative damage for individuals that cannot adjust the trade‐off between growth and self‐maintenance when fast growth occurs. This effect did not differ significantly between the types of experimental manipulation leading to the growth difference, the developmental stages at which OS was measured or the sampling methods.

The constraint‐MA provides evidence for OS as a constraint on growth as implied by the positive effect we found; increased OS (greater damage and/or reduced antioxidants) was associated with reduced growth. Both exposure to environmental stressors (e.g., inclusion of oxidized lipids in the diet, exposure to hypoxia, high stocking density) and supplementation with natural compounds (e.g., plant extracts, probiotics, herbs that have potential antioxidant properties; not specific antioxidants) produced significant positive effects. In groups exposed to environmental challenges (i.e., what we term stressors), a reduction in antioxidants might have occurred as they are utilized in neutralizing the RS that have been produced in response to the stressor. As antioxidants might improve the physiological status of cells, when they are reduced in response to these environmental challenges, as well as being lower in the unsupplemented (compared with supplemented) group, oxidative damage might increase cell necrosis and death (Costantini 2008) which could limit growth. Additionally, lower antioxidant levels might necessitate the need for a greater level of investment in antioxidant protection which could divert resources from growth.

This positive effect that provides evidence for OS as a constraint on growth was significant for natural compound but not antioxidant supplementation, and this could be due to the nature of these compounds. The antioxidants that were supplemented were of a specific type, while the natural compounds, which included plant extracts and herbs, had the potential to include a combination of numerous antioxidant molecules. Due to the synergistic effect certain antioxidants can have (Yu 1994), this might have enabled the neutralization of multiple RS molecules as well as the recycling of antioxidants that have been utilized. Therefore, this would allow reduced investment in antioxidant resources, compared with the unsupplemented group that might need to divert resources from growth. This also suggests that supplementing one type of antioxidant does not provide enough of an impact on the antioxidant status to affect growth.

All in all, the constraint‐MA implies there is a resource allocation trade‐off between growth and self‐maintenance – when greater resources are invested into maintaining oxidative status, this has a negative impact on growth, which is reduced. Moreover, our results suggest that individuals are unable to adjust this trade‐off when experimentally forced to grow faster and so are faced with OS, in terms of increased oxidative damage levels, as a cost of growth (cost‐MA). This corroborates previous work that has linked increased growth with increased damage (Nussey et al. 2009; Almroth et al. 2012; Stier et al. 2014b); where other studies have not found such a link, this might be a result of using transgenic animals that have been genetically programmed to grow faster (Rosa et al. 2008) and so may have other physiological differences making OS less notable.

The fact that the cost‐MA demonstrated that increased growth had no impact on either enzymatic or nonenzymatic antioxidants – the similar number of studies between the three biomarker groups (Fig. 1B) indicates this is not a power issue – might not be surprising given the variable results of previous studies, which have found increased growth to be associated with greater (Leggatt et al. 2007; Tobler and Sandell 2009) or lower (Kilgas et al. 2010; Almroth et al. 2012; Yengkokpam et al. 2013) antioxidant levels, as well as no change in antioxidants (Rosa et al. 2008; Larcombe et al. 2010; Geiger et al. 2011). Perhaps the variability of the antioxidant response to OS explains the lack of effect here; if increased growth caused greater RS production, levels of enzymatic and/or nonenzymatic antioxidants may be utilized in neutralizing RS and so decline or become upregulated in response to the RS therefore increase (Costantini and Verhulst 2009). The lack of clear trend in antioxidants demonstrates the complexity of the growth–OS relationship, implying that the oxidative cost of growth might not be the direct result of a diversion of resources from antioxidant protection to growth as in this case we would have expected an overall reduction in antioxidants (i.e., a positive effect). Despite debate around whether increased metabolic rate (e.g., from enhanced growth) leads to greater RS levels (Barja 2007; Fletcher et al. 2013; Stier et al. 2014b; Salin et al. 2015), our results suggest that this might be a more likely mechanism for the oxidative cost of growth.

One potential drawback to the data for cost‐MA is that it might be difficult to manipulate growth without altering other traits as well. For instance, the initial food restriction to reduce growth in the “compensatory growth” group may have reduced antioxidant status simply due to there being fewer antioxidants in the diet, which might have led to OS unrelated to growth. However, food was subsequently provided ad libitum in these groups so they had the opportunity to make up any antioxidant deficit. Likewise, enlarged broods in the “brood manipulation” group might have suffered both reduced growth and increased OS due to sibling competition but our results do not indicate this. In fact, the different experimental manipulations included in this meta‐analysis do have the potential to affect traits unrelated to growth; however, the lack of significant difference between these manipulations in how they affected growth implies their main effect was on growth and not on other traits.

Another limitation of cost‐MA is that we cannot distinguish between increased oxidative damage due to compensation and damage that might have occurred during the initial food restriction in the “compensatory growth” group because OS was only measured once in each individual. As the studies here measured OS after the period of compensatory growth, later than the initial reduction in growth rate, we could speculate the damage is due to compensation, but we cannot be sure. When considering constraint‐MA, the data might be limited by the fact that some of the growth differences observed might be unrelated to OS. For instance, exposure to environmental stressors could inhibit growth independently of the oxidative status of the cell, via alternative biochemical or physiological pathways (e.g., inhibition of cell signaling) (Inagaki et al. 2008). However, if this were the case, the association between increased OS and reduced growth that was observed in our study might not have been so apparent.

There are a number of other factors that might have affected the variation in effect sizes for both constraint‐MA and cost‐MA. Firstly, when investigating the effects of developmental stage in cost‐MA, we found no significant difference between adults, older juveniles, and early juveniles. However, one issue with including adults in our analysis is that they would come from indeterminate growing species, making it difficult to distinguish between taxonomic class and developmental stage. Furthermore, the uneven distribution of data across eight taxonomic classes that were included in these meta‐analyses makes it difficult to include phylogeny. This might mask any within‐class differences, although taxonomic class was included as a random effect to partly account for this and the variance due to taxonomic class was low.

Secondly, sampling method is important for ecological studies with a preference for nonlethal methods and this is likely to affect the tissue used in analysis (e.g., blood in nonlethal vs. organs in lethal sampling), which in turn may affect the growth–OS relationship (Brown‐Borg and Rakoczy 2003; Leggatt et al. 2007). In our meta‐analyses, most studies using nonlethal sampling were conducted in the wild, and thus, any pattern may be confounded by the collinearity of sampling method with whether the study was conducted in the laboratory or wild (for a more detailed discussion, see Appendix S3). However, we found no significant difference between lethal and nonlethal sampling for either meta‐analysis.

Lastly, the significant levels of residual heterogeneity in both meta‐analyses suggest there might be other factors affecting the variation in effect sizes, for example, sex (excluded as the majority of studies were of mixed/unknown sex) or tissue type (excluded due to the vast array of tissues sampled). While important, considerable residual heterogeneity is common in ecological meta‐analyses (Costantini and Møller 2009; Isaksson 2010; Hector and Nakagawa 2012) and could be due to random variation.

In summary, despite having a heterogeneous sample, spanning a large variety of tissues and biomarkers of OS, our results demonstrated two clear patterns. Firstly, we found OS appears to constrain growth, as greater OS (i.e., increased damage and/or reduced antioxidants) was associated with reduced growth. This could imply that lower antioxidant levels lead to resources being diverted directly from growth in a bid to enhance antioxidant protection or that a reduction in antioxidant protection leads to a deterioration of the physiological status of the cell causing oxidative damage and eventually cell necrosis and death. This would necessitate greater investment in repairing or replacing damaged cells and tissues, thus reducing that which could be put into building new cells and tissues, leading to a reduction in growth. However, the lack of significant effect, when specific antioxidants were supplemented compared with natural compounds that likely contain a mix of various antioxidant molecules, suggests that in order to have an effect on growth a combination of antioxidants must be supplemented.

Secondly, our work supports the idea of an oxidative cost of growth, at least in terms of oxidative damage to proteins, lipids, and DNA and implies individuals might not be able to adjust the growth–self‐maintenance trade‐off when forced to grow quickly. Conversely, antioxidants do not appear to have been affected by growth demonstrating the complexity of this trade‐off and we hypothesize that the oxidative cost of growth might result from an increase in metabolic rate and so RS production. This emphasizes that oxidative damage might be more relevant than antioxidants for future studies investigating the oxidative costs of growth. Overall, our results provide evidence that OS might act as both a constraint on and a cost of growth. For researchers who want to investigate this further, we recommend focussing on markers of oxidative damage rather than antioxidants and suggest that nonlethal sampling is appropriate, especially in ecological studies.

## Data Accessibility

A full list of studies included in both meta‐analyses with the effect sizes that we calculated is provided in Tables S1 and S2. Further details, including effect size variances, have been archived on Dryad.

## Conflict of Interest

None declared.

## Supporting information


**Appendix S1.** Supplementary methods.
**Appendix S2.** Supplementary results.
**Appendix S3.** Supplementary discussion.
**Figure S1.** Funnel plots for constraint‐MA and cost‐MA.
**Figure S2.** Forest plots for the treatment effect on OS (constraint‐MA) and growth (cost‐MA).
**Table S1.** Summary details for studies included in constraint‐MA.
**Table S2.** Summary details for studies included in cost‐MA.
**Table S3.** Pairwise comparisons of the different experimental approaches (cost‐MA).
