# Supplementary material for: Meta‐analysis indicates that oxidative stress is both a constraint on and a cost of growth
Source: Ecol Evol. 2016 Mar 21;6(9):2833–42. doi: 10.1002/ece3.2080 (PMC4863009; doi:10.1002/ece3.2080)
Supplement: Supplementary file 1 — Appendix S1. Supplementary methods. Appendix S2. Supplementary results. Appendix S3. Supplementary discussion. Figure S1. Funnel plots for constraint‐MA and cost‐MA. Figure S2. Forest plots for the treatment effect on OS (constraint‐MA) and growth (cost‐MA). Table S1. Summary details for studies included in constraint‐MA. Table S2. Summary details for studies included in cost‐MA. Table S3. Pairwise comparisons of the different experimental approaches (cost‐MA). [file ECE3-6-2833-s001.docx]

**Supporting information**

**Meta-analysis indicates that oxidative stress is both a constraint on and a cost of growth**

**Shona M. Smith^*a^, Ruedi G. Nager^a^ and David Costantini^a,b^**

^a^ Institute of Biodiversity, Animal Health & Comparative Medicine, Graham Kerr Building, University of Glasgow, Glasgow, G12 8QQ, UK

^b^ Department of Biology, University of Antwerp, Antwerp, 2610, Belgium

^*^ Corresponding author: s.smith.7@research.gla.ac.uk

**Appendix S1: supplementary methods**

**Data acquisition from authors**

There were 30 papers where an English copy of the text could not be accessed, in which case authors were contacted; 19 out of 30 authors responded, through which 8 more papers were obtained, the remaining 11 were not available in English. To calculate effect sizes for each paper in meta-analysis, either appropriate test statistics (e.g. t values or F ratios) or means, standard errors and sample sizes must be available. In 21 papers these values were not provided so the authors were contacted for additional information with a reminder being sent after 1 month, of which 10 responded and provided the missing information.

**Inclusion/exclusion criteria**

Inclusion and exclusion criteria were applied to select papers to include in analyses. For constraint-MA, papers were selected if they contained a comparison of growth rates between two groups that differed significantly in OS. In some cases for constraint-MA, studies did not measure OS until after the growth period was measured (e.g. if animals were sacrificed for tissue samples) so it might be questionable that this would have affected growth. However, since many redox biomarkers show intra-individual consistency, even taking into account experimental manipulations (Costantini *et al.* 2007), any differences in OS between control and experimental groups observed after the growth period would have been relatively the same before the growth period. Therefore the final OS measure was taken as a validation that OS had been significantly altered in the treatment groups (note that OS was not the response variable in the main analysis where we were interested in growth for constraint-MA). Furthermore, the time of OS measurement (i.e. before or after the growth period) was included as a factor in the statistical analysis for constraint-MA and was not found to have a significant effect. For cost-MA, papers were selected if they contained a comparison of OS levels between two groups that differed significantly in growth rate.

The following studies were excluded from both meta-analyses: those that measured reactive species (RS), since they do not provide direct evidence of OS (reactive species might be mopped up before OS arises) (Costantini, Marasco & Møller 2011); those that measured gene expression rather than activity of antioxidant molecules because we were interested in the biochemistry and not genetics of the relationship; or studies where necessary information for calculating effect size was unavailable. Where there was more than one experimental group, the two most extreme groups in terms of OS (constraint-MA) or growth rate (cost-MA) were compared.

**Standardisation of growth data**

The method by which growth rate was defined differed between studies. For most studies, growth rate was defined as the change in mass (i.e. final mass – initial mass) over time (days of experimental period). Some studies used specific growth rate (SGR), which was defined as:

$$SGR= \frac{ln(final mass)-ln(initial mass)}{number of days elapsed}$$

At times studies did not measure growth rate directly but assumed different growth rates where two groups were measured to have the same initial mass but ended with significantly different masses. The majority of studies used mass growth, except three studies in the constraint-MA that used developmental rate and one in the cost-MA that used structural size. These variations in growth measures were of no concern for either meta-analysis because standardised effect sizes for the growth difference between groups were extracted from each study where growth rates were given, thus overcoming any differences in units between studies.

**Publication bias**

Publication bias was assessed by examining funnel plots of effect size against the log of sample size for each dataset (Møller & Jennions 2001). The plot should be in the shape of a ‘funnel’ with larger variance in effect sizes at small sample sizes and a decreasing variance with increasing sample size. If only significant findings were published, one might expect there to be a ‘gap’ in the lower left of the graph, where for small samples effect sizes must be relatively large to be statistically significant. The funnel plots in the present study indicate there was no publication bias (Fig. S1). This is confirmed by the fact that sample size was not significant when added as a predictor in the models (constraint-MA: Q_M_ = 0.45, df = 1, p = 0.50; cost-MA: Q_M_ = 1.69, df = 1, p = 0.19); this method of assessing funnel plot asymmetry is recommended by Viechtbauer (2014) since it takes into account moderators (unlike the fail safe N and rank correlation).

**The effect of manipulation type on OS (constraint-MA) and growth (cost-MA)**

To investigate the relationship between growth and oxidative stress for each MA, we first had to determine whether the treatments successfully altered OS (constraint-MA) and growth (cost-MA). Note this is not a test of the main hypotheses, where we were interested in growth (constraint-MA) and OS (cost-MA) as our response variables. Hedges’ *g* values were extracted for the treatment effect on OS (constraint-MA, extracted from all studies) and on growth (cost-MA, data were unavailable for 1 study). Since the Hedges’ *g* values indicated the effectiveness of the experimental manipulation, a positive value denoted a difference in OS (constraint-MA) or growth rate (cost-MA) in the treatment group, compared with controls. The Hedges’ *g* values were the response variable in similar mixed models to that described in the main paper, with experimental manipulation as a moderator. Biomarker and sampling method were also included as moderators for constraint-MA. Lastly, model simplification was carried out as before.

**Appendix S2: supplementary results**

**The effect of manipulation type on OS (constraint-MA) and growth (cost-MA)**

All treatments significantly altered OS (constraint-MA, Fig. S2A) and growth (cost-MA, Fig.S2B). Among the categories manipulating OS (constraint-MA), all experimental manipulations had a similar sized effect on OS (Q_M_ = 0.73, df = 2, p = 0.69; see Fig. S2A for the overall treatment effect) and there were no significant differences irrespective of what biomarkers (i.e. damage, enzymatic antioxidants, non-enzymatic antioxidants; Q_M_ = 2.75, df = 2, p = 0.25) or sampling methods (i.e. lethal versus non-lethal; Q_M_ = 0.35, df = 1, p = 0.55) were measured. For cost-MA, the effect of treatment on growth depended on the type of experimental manipulation (Q_M_ = 11.20, df = 3, p = 0.01), with dietary changes producing a significantly larger effect than did brood manipulations (Table S3; Fig. S2B).

**Appendix S3: supplementary discussion**

**Did the experimental manipulations produce differences that were similar in magnitude to unmanipulated levels?**

All three categories of growth manipulation in cost-MA (compensatory growth, brood manipulation and dietary changes) produced growth differences that were similar in magnitude to those occurring in the unmanipulated group (manipulation category ‘None’, see Table 1 in the main paper for full description), since the effects of treatment on growth did not differ significantly between manipulated and unmanipulated groups (Table S3). However, given that there were no observational studies in which differences in OS between unmanipulated groups had been compared, it is difficult to tell whether the treatments altering OS produced comparable differences with unmanipulated groups (constraint-MA); as was determined for the treatment effects on growth in cost-MA. This could imply that the experimental manipulations led to unrealistic differences in OS. However strong effects of OS on growth were reported for studies in which species were exposed to stressors that would be encountered naturally (e.g. pond desiccation; Hedges’ *g* = 2.06 ± 1.13 (Gomez-Mestre, Kulkarni & Buchholz 2013)) or that are potentially encountered in farming situations (e.g. hypoxia in fish; Hedges’ *g* = -1.41 ± 1.35 (Filho *et al.* 2005); high stocking density in broiler chickens; Hedges’ *g* = 2.75 ± 1.73 (Simitzis *et al.* 2012)). Therefore the results are still considered relevant for assessing how OS can affect growth rate, and might prove to be a useful consideration for animal welfare issues.

**The issue with sampling method**

The high collinearity (cost-MA) of sampling method with whether the study was conducted in the laboratory or wild (excluded from analysis) makes it difficult to determine the underlying cause of any differences between lethal and non-lethal sampling. The lack of environmental challenges that reduce fitness in the laboratory might mean effects are only evident in field studies of wild animals. Since field studies tended to use non-lethal sampling, a greater effect here could be wrongly interpreted as a greater effect with non-lethal sampling. Yet we found no significant difference between the two sampling methods for either constraint-MA or cost-MA, which might suggest the tissues sampled by non-lethal methods are sufficient for investigating the growth-OS relationship. The constraint-MA included only 3 studies conducted in the wild so it is difficult to determine if this collinearity would be an issue and when the analysis was repeated excluding these, similar results were obtained – there was no significant difference between lethal and non-lethal sampling.

**References**

Alonso-Álvarez, C., Bertrand, S., Faivre, B. & Sorci, G. (2007) Increased susceptibility to oxidative damage as a cost of accelerated somatic growth in zebra finches. *Functional Ecology*, **21**, 873–879.

de Ayala, R.M., Martinelli, R. & Saino, N. (2006) Vitamin E supplementation enhances growth and condition of nestling barn swallows (*Hirundo rustica*). *Behavioral Ecology and Sociobiology*, **60**, 619–630.

Aziza, A.E., Awadin, W.F. & Orma, A.M. (2013) Effect of dietary substitution of cod liver oil by vegetable oils on growth performance, body composition, lipid peroxidation, liver and muscle histopathological state in *Nile tilapia (Oreochromis niloticus)*. *Journal of Fisheries and Aquaculture*, **4**, 87–94.

De Block, M. & Stoks, R. (2008) Compensatory growth and oxidative stress in a damselfly. *Proceedings of the Royal Society B*, **275**, 781–785.

Can, E., Kurtoğlu, İ.Z., Benzer, F., Erişir, M., Kocabaş, M., Kızak, V., Kayım, M. & Çelik, H.T. (2012) The effects of different dosage of kefir with different durations on growth performances and antioxidant system in the blood and liver tissues of Çoruh trout (*Salmo coruhensis*). *Turkish Journal of Fisheries and Aquatic Sciences*, **12**, 277–283.

Chen, Y.J., Liu, Y.J., Tian, L.X., Niu, J., Liang, G.Y., Yang, H.J., Yuan, Y. & Zhang, Y.Q. (2013) Effect of dietary vitamin E and selenium supplementation on growth, body composition, and antioxidant defense mechanism in juvenile largemouth bass (*Micropterus salmoides*) fed oxidized fish oil. *Fish Physiology and Biochemistry*, **39**, 593–604.

Chen, Y.J., Liu, Y.J., Yang, H.J., Yuan, Y., Liu, F.J., Tian, L.X., Liang, G.Y. & Yuan, R.M. (2012) Effect of dietary oxidized fish oil on growth performance, body composition, antioxidant defence mechanism and liver histology of juvenile largemouth bass *Micropterus salmoides*. *Aquaculture Nutrition*, **18**, 321–331.

Chien, Y.H. & Shiau, W.C. (2005) The effects of dietary supplementation of algae and synthetic astaxanthin on body astaxanthin, survival, growth, and low dissolved oxygen stress resistance of kuruma prawn, *Marsupenaeus japonicus* Bate. *Journal of Experimental Marine Biology and Ecology*, **318**, 201–211.

Cinar, M., Yildirim, E., Yigit, A.A., Yalcinkaya, I., Duru, O., Kisa, U. & Atmaca, N. (2014) Effects of dietary supplementation with vitamin C and vitamin E and their combination on growth performance, some biochemical parameters, and oxidative stress induced by copper toxicity in broilers. *Biological Trace Element Research*, **158**, 186–96.

Costantini, D. (2010) Effects of diet quality on growth pattern, serum oxidative status, and corticosterone in Pigeons (*Columba livia*). *Canadian Journal of Zoology*, **88**, 795–802.

Costantini, D., Coluzza, C., Fanfani, A. & Dell’Omo, G. (2007) Effects of carotenoid supplementation on colour expression, oxidative stress and body mass in rehabilitated captive adult kestrels (Falco tinnunculus). *Journal of comparative physiology. B, Biochemical, systemic, and environmental physiology*, **177**, 723–31.

Costantini, D., Marasco, V. & Møller, A.P. (2011) A meta-analysis of glucocorticoids as modulators of oxidative stress in vertebrates. *Journal of Comparative Physiology B: Biochemical, Systemic and Environmental Physiology*, **181**, 447–456.

Díaz-Cruz, A., Serret, M., Ramírez, G., Avila, E., Guinzberg, R. & Piña, E. (2003) Prophylactic action of lipoic acid on oxidative stress and growth performance in broilers at risk of developing ascites syndrome. *Avian Pathology*, **32**, 645–653.

Djuric, Z., Lewis, S.M., Lu, M.H., Mayhugh, M., Tang, N. & Hart, R.W. (2009) Effect of varying dietary fat levels on rat growth and oxidative DNA damage. *Nutrition and Cancer*, **39**, 214–219.

Dong, G.F., Yang, Y.O., Song, X.M., Yu, L., Zhao, T.T., Huang, G.L., Hu, Z.J. & Zhang, J.L. (2013) Comparative effects of dietary supplementation with maggot meal and soybean meal in gibel carp (*Carassius auratus gibelio*) and darkbarbel catfish (*Pelteobagrus vachelli*): growth performance and antioxidant responses. *Aquaculture Nutrition*, **19**, 543–554.

Filho, D.W., Torres, M.A., Zaniboni-Filho, E. & Pedrosa, R.C. (2005) Effect of different oxygen tensions on weight gain, feed conversion, and antioxidant status in piapara, *Leporinus elongatus* (Valenciennes, 1847). *Aquaculture*, **244**, 349–357.

Fontagné, S., Bazin, D., Brèque, J., Vachot, C., Bernarde, C., Rouault, T. & Bergot, P. (2006) Effects of dietary oxidized lipid and vitamin A on the early development and antioxidant status of Siberian sturgeon (*Acipenser baeri*) larvae. *Aquaculture*, **257**, 400–411.

Fontagné, S., Lataillade, E., Brèque, J. & Kaushik, S. (2008) Lipid peroxidative stress and antioxidant defence status during ontogeny of rainbow trout (*Oncorhynchus mykiss*). *The British Journal of Nutrition*, **100**, 102–111.

Gao, J., Koshio, S., Ishikawa, M., Yokoyama, S. & Mamauag, R.E.P. (2014a) Interactive effects of vitamin C and E supplementation on growth performance, fatty acid composition and reduction of oxidative stress in juvenile Japanese flounder *Paralichthys olivaceus* fed dietary oxidized fish oil. *Aquaculture*, **422-423**, 84–90.

Gao, J., Koshio, S., Ishikawa, M., Yokoyama, S., Mamauag, R.E.P. & Han, Y. (2012) Effects of dietary oxidized fish oil with vitamin E supplementation on growth performance and reduction of lipid peroxidation in tissues and blood of red sea bream *Pagrus major*. *Aquaculture*, **356-357**, 73–79.

Gao, J., Koshio, S., Ishikawa, M., Yokoyama, S., Nguyen, B.T. & Mamauag, R.E. (2013a) Effect of dietary oxidized fish oil and vitamin C supplementation on growth performance and reduction of oxidative stress in Red Sea Bream *Pagrus major*. *Aquaculture Nutrition*, **19**, 35–44.

Gao, J., Koshio, S., Ishikawa, M., Yokoyama, S., Nose, D. & Ren, T. (2013b) Interactive effects of vitamin C and E supplementation on growth, fatty acid composition, and lipid peroxidation of sea cucumber, *Apostichopus japonicus*, fed with dietary oxidized fish oil. *Journal of the World Aquaculture Society*, **44**, 536–546.

Gao, J., Koshio, S., Wang, W., Li, Y., Huang, S. & Cao, X. (2014b) Effects of dietary phospholipid levels on growth performance, fatty acid composition and antioxidant responses of Dojo loach *Misgurnus anguillicaudatus* larvae. *Aquaculture*, **426-427**, 304–309.

Geiger, S., Le Vaillant, M., Lebard, T., Reichert, S., Stier, A., Le Maho, Y. & Criscuolo, F. (2011) Catching-up but telomere loss: half-opening the black box of growth and ageing trade-off in wild king penguin chicks. *Molecular Ecology*, **21**, 1500–1510.

Gomez-Mestre, I., Kulkarni, S. & Buchholz, D.R. (2013) Mechanisms and consequences of developmental acceleration in tadpoles responding to pond drying. *PloS One*, **8**, e84266.

Guerra, C., Zenteno-Savín, T., Maeda-Martínez, A.N., Philipp, E.E.R. & Abele, D. (2012) Changes in oxidative stress parameters in relation to age, growth and reproduction in the short-lived catarina scallop *Argopecten ventricosus* reared in its natural environment. *Comparative Biochemistry and Physiology A: Molecular & Integrative Physiology*, **162**, 421–430.

Hargitai, R., Costantini, D., Moskát, C., Bán, M., Muriel, J. & Hauber, M.E. (2012) Variation in plasma oxidative status and testosterone level in relation to egg-eviction effort and age of brood-parasitic common cuckoo nestlings. *The Condor*, **114**, 782–791.

Hisar, O., Yanik, T., Kocaman, E.M., Arslan, M., Slukvin, A. & Goncharova, R. (2012) Effects of diludine supplementation on growth performance, liver antioxidant enzyme activities and muscular trace elements of rainbow trout (*Oncorhynchus mykiss*) juveniles at a low water temperature. *Aquaculture Nutrition*, **18**, 211–219.

Huang, C.H., Higgs, D.A., Balfry, S.K. & Devlin, R.H. (2004) Effect of dietary vitamin E level on growth, tissue lipid peroxidation, and erythrocyte fragility of transgenic coho salmon, *Oncorhynchus kisutch*. *Comparative Biochemistry and Physiology A: Molecular & Integrative Physiology*, **139**, 199–204.

Huang, C.H. & Huang, S.L. (2004) Effect of dietary vitamin E on growth, tissue lipid peroxidation, and liver glutathione level of juvenile hybrid tilapia, *Oreochromis niloticus*×*O. aureus*, fed oxidized oil. *Aquaculture*, **237**, 381–389.

Huang, C.H. & Lin, W.Y. (2004) Effects of dietary vitamin E level on growth and tissue lipid peroxidation of soft-shelled turtle, *Pelodiscus sinensis* (Wiegmann). *Aquaculture Research*, **35**, 948–954.

Ijiri, D., Nakamura, S., Tatsugawa, K., Ijiri, S. & Ohtsuka, A. (2013) Effects of feeding dried concentrated rice-washing water on growth performance and skeletal muscle lipid peroxidation in broiler chickens. *The Journal of Poultry Science*, **50**, 370–374.

Kader, M.A., Koshio, S., Ishikawa, M., Yokoyama, S., Bulbul, M., Honda, Y., Mamauag, R.E. & Laining, A. (2010) Growth, nutrient utilization, oxidative condition, and element composition of juvenile red sea bream *Pagrus major* fed with fermented soybean meal and scallop by-product blend as fishmeal replacement. *Fisheries Science*, **77**, 119–128.

Kato, N., Kawai, K. & Yoshida, A. (1981) Effect of dietary level of ascorbic acid on the growth, hepatic lipid peroxidation, and serum lipids in guinea piges fed polychlorinated biphenyls. *Journal of Nutrition*, **111**, 1727–1733.

Keleştemur, G.T. & Seven, İ. (2013) Effects of dietary propolis and vitamin E on growth performance and antioxidant status in juvenile rainbow trout (*Oncorhynchus mykiss*) under different flow rate. *Aquaculture Research*, **44**, 1120–1131.

Khempaka, S., Pudpila, U. & Molee, W. (2013) Effect of dried peppermint (*Mentha cordifolia*) on growth performance, nutrient digestibility, carcass traits, antioxidant properties, and ammonia production in broilers. *Journal of Applied Poultry Research*, **22**, 904–912.

Kilgas, P., Tilgar, V., Külavee, R., Saks, L., Hõrak, P. & Mänd, R. (2010) Antioxidant protection, immune function and growth of nestling great tits *Parus major* in relation to within-brood hierarchy. *Comparative Biochemistry and Physiology B: Biochemistry & Molecular Biology*, **157**, 288–293.

Kütter, M.T., Monserrat, J.M., Primel, E.G., Caldas, S.S. & Tesser, M.B. (2012) Effects of dietary α-lipoic acid on growth, body composition and antioxidant status in the Plata pompano *Trachinotus marginatus* (Pisces, Carangidae). *Aquaculture*, **368-369**, 29–35.

Langley-Evans, S.C. & Sculley, D. V. (2006) The association between birthweight and longevity in the rat is complex and modulated by maternal protein intake during fetal life. *FEBS Letters*, **580**, 4150–4153.

Lee, J.S., Cheng, H., Damte, D., Lee, S.J., Kim, J.C., Rhee, M.H., Suh, J.W. & Park, S.C. (2013) Effects of dietary supplementation of *Lactobacillus pentosus* PL11 on the growth performance, immune and antioxidant systems of Japanese eel *Anguilla japonica* challenged with *Edwardsiella tarda*. *Fish & Shellfish Immunology*, **34**, 756–761.

Lee, K.J. & Dabrowski, K. (2003) Interaction between vitamins C and E affects their tissue concentrations, growth, lipid oxidation, and deficiency symptoms in yellow perch (*Perca flavescens*). *The British Journal of Nutrition*, **89**, 589–596.

Li, Z.H., Xie, S., Wang, J.X., Sales, J., Li, P. & Chen, D.Q. (2009) Effect of intermittent starvation on growth and some antioxidant indexes of *Macrobrachium nipponense* (De Haan). *Aquaculture Research*, **40**, 526–532.

Liu, H.W., Dong, X.F., Tong, J.M. & Zhang, Q. (2010) Alfalfa polysaccharides improve the growth performance and antioxidant status of heat-stressed rabbits. *Livestock Science*, **131**, 88–93.

Lu, T., Harper, A.F., Zhao, J. & Dalloul, R.A. (2014) Effects of a dietary antioxidant blend and vitamin E on growth performance, oxidative status, and meat quality in broiler chickens fed a diet high in oxidants. *Poultry Science*, **93**, 1649–1657.

Luo, Z., Tan, X.Y., Li, X.D. & Yin, G.J. (2012) Effect of dietary arachidonic acid levels on growth performance, hepatic fatty acid profile, intermediary metabolism and antioxidant responses for juvenile *Synechogobius hasta*. *Aquaculture Nutrition*, **18**, 340–348.

Ma, J.J., Xu, Z.R., Shao, Q.J., Xu, J.Z., Hung, S.S.O., Hu, W.L. & Zhuo, L.Y. (2008) Effect of dietary supplemental l-carnitine on growth performance, body composition and antioxidant status in juvenile black sea bream, *Sparus macrocephalus*. *Aquaculture Nutrition*, **14**, 464–471.

Marri, V. & Richner, H. (2014) Differential effects of vitamins E and C and carotenoids on growth, resistance to oxidative stress, fledging success and plumage colouration in wild great tits. *Journal of Experimental Biology*, **217**, 1478–1484.

Møller, A.P. & Jennions, M.D. (2001) Testing and adjusting for publication bias. *Trends in Ecology & Evolution*, **16**, 580–586.

Mustafa, S.A., Al-Subiai, S.N., Davies, S.J. & Jha, A.N. (2011) Hypoxia-induced oxidative DNA damage links with higher level biological effects including specific growth rate in common carp, *Cyprinus carpio* L. *Ecotoxicology*, **20**, 1455–1466.

Nain, S., Ling, B., Bandy, B., Alcorn, J., Wojnarowicz, C., Laarveld, B. & Olkowski, A.A. (2008) The role of oxidative stress in the development of congestive heart failure in a chicken genotype selected for rapid growth. *Avian Pathology*, **37**, 367–373.

Niu, J., Li, C.H., Liu, Y.J., Tian, L.X., Chen, X., Huang, Z. & Lin, H.Z. (2012) Dietary values of astaxanthin and canthaxanthin in *Penaeus monodon* in the presence and absence of cholesterol supplementation: effect on growth, nutrient digestibility and tissue carotenoid composition. *The British Journal of Nutrition*, **108**, 80–91.

Niu, J., Lin, H.Z., Jiang, S.G., Chen, X., Wu, K.C., Liu, Y.J., Wang, S. & Tian, L.X. (2013) Comparison of effect of chitin, chitosan, chitosan oligosaccharide and N-acetyl-d-glucosamine on growth performance, antioxidant defenses and oxidative stress status of *Penaeus monodon*. *Aquaculture*, **372-375**, 1–8.

Noguera, J.C., Lores, M., Alonso-Álvarez, C. & Velando, A. (2011) Thrifty development: early-life diet restriction reduces oxidative damage during later growth. *Functional Ecology*, **25**, 1144–1153.

Ohtsuka, A., Ohtani, T., Horiguchi, H., Kojima, H. & Hayashi, K. (1998) Vitamin E reduces glucocorticoid-induced growth inhibition and lipid peroxidation in rats. *Journal of Nutritional Science and Vitaminology*, **44**, 237–247.

Onderci, M., Sahin, K., Sahin, N., Cikim, G., Vijaya, J. & Kucuk, O. (2005) Effects of dietary combination of chromium and biotin on growth performance, carcass characteristics, and oxidative stress markers in heat-distressed Japanese quail. *Biological Trace Element Research*, **106**, 165–176.

Özlüer-Hunt, A., Berköz, M., Özkan, F., Yalin, S., Erçen, Z., Erdoğan, E. & Gündüz, S.G. (2011) Effect of mannan oligosaccharide on growth, body composition, and antioxidant enzyme activity of tilapia (*Oreochromis niloticus*). *The Israeli Journal of Aquaculture*, **63**, 1–8.

Peng, S., Chen, L., Qin, J.G., Hou, J., Yu, N., Long, Z., Li, E. & Ye, J. (2009) Effects of dietary vitamin E supplementation on growth performance, lipid peroxidation and tissue fatty acid composition of black sea bream (*Acanthopagrus schlegeli*) fed oxidized fish oil. *Aquaculture Nutrition*, **15**, 329–337.

Radhakrishnan, S., Bhavan, P.S., Seenivasan, C., Shanthi, R. & Poongodi, R. (2013) Influence of medicinal herbs (*Alteranthera sessilis*, *Eclipta alba* and *Cissus quadrangularis*) on growth and biochemical parameters of the freshwater prawn *Macrobrachium rosenbergii*. *Aquaculture International*, **22**, 551–572.

Roark, A.M., Bjorndal, K.A., Bolten, A.B. & Leeuwenburgh, C. (2009) Biochemical indices as correlates of recent growth in juvenile green turtles (*Chelonia mydas*). *Journal of Experimental Marine Biology and Ecology*, **376**, 59–67.

Saïdi, S.A., Azaza, M.S., Abdelmouleh, A., Pelt, J. Van, Kraïem, M.M. & El-Feki, A. (2010) The use of tuna industry waste in the practical diets of juvenile Nile tilapia (*Oreochromis niloticus*, L.): effect on growth performance, nutrient digestibility and oxidative status. *Aquaculture Research*, **41**, 1875–1886.

Salin, K., Luquet, E., Rey, B., Roussel, D. & Voituron, Y. (2012) Alteration of mitochondrial efficiency affects oxidative balance, development and growth in frog (*Rana temporaria*) tadpoles. *Journal of Experimental Biology*, **215**, 863–869.

Salomons, H.M. (2009) *Fighting for Fitness: Telomeres, Oxidative Stress and Life History Trade-Offs in a Colonial Corvid (Chapter 3)*. Ph.D. Thesis, University of Groningen, The Netherlands.

Savary-Auzeloux, I., Durand, D., Gruffat, D., Bauchart, D. & Ortigues-Marty, I. (2008) Food restriction and refeeding in lambs influence muscle antioxidant status. *Animal*, **2**, 738–745.

Shen, W.Y., Fu, L.L., Li, W.F. & Zhu, Y.R. (2010) Effect of dietary supplementation with *Bacillus subtilis* on the growth, performance, immune response and antioxidant activities of the shrimp (*Litopenaeus vannamei*). *Aquaculture Research*, **41**, 1691–1698.

Simitzis, P.E., Kalogeraki, E., Goliomytis, M., Charismiadou, M.A., Triantaphyllopoulos, K., Ayoutanti, A., Niforou, K., Hager-Theodorides, A.L. & Deligeorgis, S.G. (2012) Impact of stocking density on broiler growth performance, meat characteristics, behavioural components and indicators of physiological and oxidative stress. *British Poultry Science*, **53**, 721–730.

Sitjà-Bobadilla, A., Peña-Llopis, S., Gómez-Requeni, P., Médale, F., Kaushik, S. & Pérez-Sánchez, J. (2005) Effect of fish meal replacement by plant protein sources on non-specific defence mechanisms and oxidative stress in gilthead sea bream (*Sparus aurata*). *Aquaculture*, **249**, 387–400.

Stier, A., Delestrade, A., Zahn, S., Arrivé, M., Criscuolo, F. & Massemin-Challet, S. (2014) Elevation impacts the balance between growth and oxidative stress in coal tits. *Oecologia*, **175**, 791–800.

Sun, H., Wang, W., Geng, L., Chen, Y. & Yang, Z. (2013) *In situ* studies on growth, oxidative stress responses, and gene expression of juvenile bighead carp (*Hypophthalmichthys nobilis*) to eutrophic lake water dominated by cyanobacterial blooms. *Chemosphere*, **93**, 421–427.

Taniguchi, N., Ohtsuka, A. & Hayashi, K. (1999) Effect of dietary corticosterone and vitamin E on growth and oxidative stress in broiler chickens. *Animal Science Journal*, **70**, 195–200.

Tarry-Adkins, J.L., Martin-Gronert, M.S., Chen, J.H., Cripps, R.L. & Ozanne, S.E. (2008) Maternal diet influences DNA damage, aortic telomere length, oxidative stress, and antioxidant defense capacity in rats. *The FASEB Journal*, **22**, 2037–2044.

Thiamhirunsopit, K., Phisalaphong, C., Boonkird, S. & Kijparkorn, S. (2014) Effect of chili meal (*Capsicum frutescens* LINN.) on growth performance, stress index, lipid peroxidation and ileal nutrient digestibility in broilers reared under high stocking density condition. *Animal Feed Science and Technology*, **192**, 90–100.

Viechtbauer, W. (2014) Metafor: package updates. URL http://www.metafor-project.org/doku.php/updates [accessed 9 September 2014]

Wang, J.P., Chi, F. & Kim, I.H. (2012a) Effects of montmorillonite clay on growth performance, nutrient digestibility, vulva size, faecal microflora, and oxidative stress in weaning gilts challenged with zearalenone. *Animal Feed Science and Technology*, **178**, 158–166.

Wang, Y.J., Chien, Y.H. & Pan, C.H. (2006) Effects of dietary supplementation of carotenoids on survival, growth, pigmentation, and antioxidant capacity of characins, *Hyphessobrycon callistus*. *Aquaculture*, **261**, 641–648.

Wang, J., Ji, H.F., Wang, S.X., Zhang, D.Y., Liu, H., Shan, D.C. & Wang, Y.M. (2012b) *Lactobacillus plantarum* ZLP001: *in vitro* assessment of antioxidant capacity and effect on growth performance and antioxidant status in weaning piglets. *Asian-Australasian Journal of Animal Sciences*, **25**, 1153–1158.

Wang, X., Kim, K. & Bai, S.C. (2002) Effects of different dietary levels of L-ascorbyl-2-polyphosphate on growth and tissue vitamin C concentrations in juvenile olive flounder, *Paralichthys olivaceus* (Temminck et Schlegel). *Aquaculture Research*, **33**, 261–267.

Wang, H., Yang, W., Wang, Y., Yang, Z. & Cui, Y. (2011) The study on the effects of chinese herbal mixtures on growth, activity of post-ruminal digestive enzymes and serum antioxidant status of beef cattle. *Agricultural Sciences in China*, **10**, 448–455.

Wang, J., Zhang, D., Sun, Y., Wang, S., Li, P., Gatlin, D.M. & Zhang, L. (2014) Effect of a dairy-yeast prebiotic (GroBiotic ® -A) on growth performance, body composition, antioxidant capacity and immune functions of juvenile starry flounder (*Platichthys stellatus*). *Aquaculture Research*, DOI: 10.1111/are.12501.

Xu, W.J. & Pan, L.Q. (2014) Evaluation of dietary protein level on selected parameters of immune and antioxidant systems, and growth performance of juvenile *Litopenaeus vannamei* reared in zero-water exchange biofloc-based culture tanks. *Aquaculture*, **426-427**, 181–188.

Yang, S.P., Wu, Z.H., Jian, J.C. & Zhang, X.Z. (2010) Effect of marine red yeast *Rhodosporidium paludigenum* on growth and antioxidant competence of *Litopenaeus vannamei*. *Aquaculture*, **309**, 62–65.

Yengkokpam, S., Debnath, D., Pal, A.K., Sahu, N.P., Jain, K.K., Norouzitallab, P. & Baruah, K. (2013) Short-term periodic feed deprivation in *Labeo rohita* fingerlings: effect on the activities of digestive, metabolic and anti-oxidative enzymes. *Aquaculture*, **412-413**, 186–192.

Yi, D., Gu, L., Ding, B., Li, M., Hou, Y., Wang, L. & Gong, J. (2012) Effects of dietary silymarin supplementation on growth performance and oxidative status in *Carassius auratus gibelio*. *Journal of Animal and Veterinary Advances*, **11**, 3399–3404.

Yurtseven, S., Çetin, M., Şengül, T. & Sögüt, B. (2008) Effect of sage extract (*Salvia officinalis*) on growth performance, blood parameters, oxidative stress and DNA damage in partridges. *South African Journal of Animal Science*, **38**, 145–152.

Zhang, W., Chen, Q., Mai, K., Xu, W., Wang, X. & Liufu, Z. (2010) Effects of dietary α-lipoic acid on the growth and antioxidative responses of juvenile abalone *Haliotis discus hannai* Ino. *Aquaculture Research*, **41**, e781–e787.

Zhang, J., Liu, Y.J., Tian, L.X., Yang, H.J., Liang, G.Y., Yue, Y.R. & Xu, D.H. (2013a) Effects of dietary astaxanthin on growth, antioxidant capacity and gene expression in Pacific white shrimp *Litopenaeus vannamei*. *Aquaculture Nutrition*, **19**, 917–927.

Zhang, G.G., Yang, Z.B., Wang, Y. & Yang, W.R. (2013b) Effects of *Astragalus membranaceus* root processed to different particle sizes on growth performance, antioxidant status, and serum metabolites of broiler chickens. *Poultry Science*, **92**, 178–183.

Zhang, G.F., Yang, Z.B., Wang, Y., Yang, W.R., Jiang, S.Z. & Gai, G.S. (2009) Effects of ginger root (*Zingiber officinale*) processed to different particle sizes on growth performance, antioxidant status, and serum metabolites of broiler chickens. *Poultry Science*, **88**, 2159–2166.

Zheng, J., Chen, L.L., Zhang, H.H., Hu, X., Kong, W. & Hu, D. (2012) Resveratrol improves insulin resistance of catch-up growth by increasing mitochondrial complexes and antioxidant function in skeletal muscle. *Metabolism: Clinical and Experimental*, **61**, 954–965.

Zheng, L., Ma, Y.E., Gu, L.Y., Yuan, D., Shi, M.L., Guo, X.Y. & Zhan, X.A. (2013) Growth performance, antioxidant status, and nonspecific immunity in broilers under different lighting regimens. *Journal of Applied Poultry Research*, **22**, 798–807.

Zheng, Z.L., Tan, J.Y.W., Liu, H.Y., Zhou, X.H., Xiang, X. & Wang, K.Y. (2009) Evaluation of oregano essential oil (*Origanum heracleoticum* L.) on growth, antioxidant effect and resistance against *Aeromonas hydrophila* in channel catfish (*Ictalurus punctatus*). *Aquaculture*, **292**, 214–218.

Zhou, Q.C., Wang, L.G., Wang, H.L., Wang, T., Elmada, C.Z. & Xie, F.J. (2013) Dietary vitamin E could improve growth performance, lipid peroxidation and non-specific immune responses for juvenile cobia (*Rachycentron canadum*). *Aquaculture Nutrition*, **19**, 421–429.

Table S1 Summary details for studies included in constraint-MA investigating the effects of OS on growth rate.

| **Species** | **Taxonomic Class** | **Experimental Manipulation** | **Biological Matrix** | **Sampling Method** | **Biomarker** | **Biomarker Category** | **N** | **Hedges' g** | **Authors** |
| --- | --- | --- | --- | --- | --- | --- | --- | --- | --- |
| *Acipenser baerii* | Actinopterygii | stressor exposure | whole body | lethal | isoprostanes | damage | 40 | 3.75 | (Fontagné *et al.* 2006) |
| *Acipenser baerii* | Actinopterygii | stressor exposure | whole body | lethal | TBARS | damage | 40 | 3.75 | (Fontagné *et al.* 2006) |
| *Acipenser baerii* | Actinopterygii | stressor exposure | whole body | lethal | Se-GPX | enzyme | 40 | 3.75 | (Fontagné *et al.* 2006) |
| *Alectoris chukar* | Aves | natural compound supplementation | blood | non-lethal | DNA damage (Comet) | damage | 6 | 0.63 | (Yurtseven *et al.* 2008) |
| *Anguilla japonica* | Actinopterygii | natural compound supplementation | liver | lethal | CAT | enzyme | 6 | 5.58 | (Lee *et al.* 2013) |
| *Anguilla japonica* | Actinopterygii | natural compound supplementation | liver | lethal | SOD | enzyme | 6 | 5.58 | (Lee *et al.* 2013) |
| *Apostichopus japonicus* | Holothuroidea | stressor exposure | whole body | lethal | TBARS | damage | 6 | 5.78 | (Gao *et al.* 2013a) |
| *Apostichopus japonicus* | Holothuroidea | antioxidant supplementation | whole body | lethal | TBARS | damage | 6 | 0.8 | (Gao *et al.* 2013a) |
| *Apostichopus japonicus* | Holothuroidea | antioxidant supplementation | whole body | lethal | vitamin E | non-enzymatic | 6 | 0.8 | (Gao *et al.* 2013a) |
| *Apostichopus japonicus* | Holothuroidea | stressor exposure | whole body | lethal | vitamin E | non-enzymatic | 6 | 5.78 | (Gao *et al.* 2013a) |
| *Bos taurus* | Mammalia | natural compound supplementation | serum | non-lethal | MDA | damage | 8 | 0.17 | (Wang *et al.* 2011) |
| *Bos taurus* | Mammalia | natural compound supplementation | serum | non-lethal | SOD | enzyme | 8 | 0.17 | (Wang *et al.* 2011) |
| *Carassius auratus gibelio* | Actinopterygii | natural compound supplementation | liver | lethal | MDA | damage | 30 | 0.32 | (Yi *et al.* 2012) |
| *Carassius auratus gibelio* | Actinopterygii | natural compound supplementation | serum | non-lethal | MDA | damage | 30 | 0.32 | (Yi *et al.* 2012) |
| *Cavia porcellus* | Mammalia | antioxidant supplementation | liver | lethal | vitamin C | non-enzymatic | 12 | 1.25 | (Kato, Kawai & Yoshida 1981) |
| *Coturnix japonica* | Aves | stressor exposure | liver | lethal | MDA | damage | 20 | 0.96 | (Onderci *et al.* 2005) |
| *Coturnix japonica* | Aves | stressor exposure | muscle | lethal | MDA | damage | 20 | 0.96 | (Onderci *et al.* 2005) |
| *Coturnix japonica* | Aves | stressor exposure | serum | non-lethal | MDA | damage | 20 | 0.96 | (Onderci *et al.* 2005) |
| *Coturnix japonica* | Aves | stressor exposure | serum | non-lethal | vitamin C | non-enzymatic | 20 | 0.96 | (Onderci *et al.* 2005) |
| *Coturnix japonica* | Aves | stressor exposure | serum | non-lethal | vitamin E | non-enzymatic | 20 | 0.96 | (Onderci *et al.* 2005) |
| *Cyprinus carpio* | Actinopterygii | stressor exposure | liver | lethal | GPX | enzyme | 12 | 1.35 | (Mustafa *et al.* 2011) |
| *Gallus gallus* | Aves | antioxidant supplementation | plasma | non-lethal | vitamin A | non-enzymatic | 18 | -0.22 | (Cinar *et al.* 2014) |
| *Gallus gallus* | Aves | antioxidant supplementation | blood | non-lethal | GPX | enzyme | 18 | 0.22 | (Cinar *et al.* 2014) |
| *Gallus gallus* | Aves | antioxidant supplementation | plasma | non-lethal | vitamin E | non-enzymatic | 18 | 0.22 | (Cinar *et al.* 2014) |
| *Gallus gallus* | Aves | natural compound supplementation | liver | lethal | TBARS | damage | 516 | 0.06 | (Díaz-Cruz *et al.* 2003) |
| *Gallus gallus* | Aves | natural compound supplementation | liver | lethal | tGSH | non-enzymatic | 516 | 0.06 | (Díaz-Cruz *et al.* 2003) |
| *Gallus gallus* | Aves | natural compound supplementation | muscle | lethal | MDA | damage | 14 | -0.17 | (Ijiri *et al.* 2013) |
| *Gallus gallus* | Aves | natural compound supplementation | plasma | non-lethal | TBARS | damage | 8 | 0.03 | (Khempaka, Pudpila & Molee 2013) |
| *Gallus gallus* | Aves | antioxidant supplementation | plasma | non-lethal | TBARS | damage | 20 | 0.27 | (Lu *et al.* 2014) |
| *Gallus gallus* | Aves | stressor exposure | heart | lethal | GSH:GSSG | non-enzymatic | 12 | 2.75 | (Simitzis *et al.* 2012) |
| *Gallus gallus* | Aves | stressor exposure | heart | lethal | tGSH | non-enzymatic | 12 | 2.75 | (Simitzis *et al.* 2012) |
| *Gallus gallus* | Aves | stressor exposure | liver | lethal | GSH:GSSG | non-enzymatic | 12 | 2.75 | (Simitzis *et al.* 2012) |
| *Gallus gallus* | Aves | stressor exposure | liver | lethal | tGSH | non-enzymatic | 12 | 2.75 | (Simitzis *et al.* 2012) |
| *Gallus gallus* | Aves | stressor exposure | spleen | lethal | GSH:GSSG | non-enzymatic | 12 | 2.75 | (Simitzis *et al.* 2012) |
| *Gallus gallus* | Aves | stressor exposure | testes | lethal | GSH:GSSG | non-enzymatic | 12 | 2.75 | (Simitzis *et al.* 2012) |
| *Gallus gallus* | Aves | stressor exposure | testes | lethal | tGSH | non-enzymatic | 12 | 2.75 | (Simitzis *et al.* 2012) |
| *Gallus gallus* | Aves | stressor exposure | liver | lethal | MDA | damage | 12 | 1.85 | (Taniguchi, Ohtsuka & Hayashi 1999) |
| *Gallus gallus* | Aves | antioxidant supplementation | liver | lethal | MDA | damage | 12 | 1.51 | (Taniguchi *et al.* 1999) |
| *Gallus gallus* | Aves | stressor exposure | plasma | non-lethal | MDA | damage | 8 | 2.78 | (Thiamhirunsopit *et al.* 2014) |
| *Gallus gallus* | Aves | natural compound supplementation | plasma | non-lethal | MDA | damage | 8 | -0.26 | (Thiamhirunsopit *et al.* 2014) |
| *Gallus gallus* | Aves | natural compound supplementation | serum | non-lethal | MDA | damage | 8 | 0.4 | (Zhang *et al.* 2013a) |
| *Gallus gallus* | Aves | natural compound supplementation | serum | non-lethal | GPX | enzyme | 8 | 0.4 | (Zhang *et al.* 2013a) |
| *Gallus gallus* | Aves | natural compound supplementation | serum | non-lethal | SOD | enzyme | 8 | 0.4 | (Zhang *et al.* 2013a) |
| *Gallus gallus* | Aves | natural compound supplementation | serum | non-lethal | MDA | damage | 8 | 0.72 | (Zhang *et al.* 2009) |
| *Gallus gallus* | Aves | natural compound supplementation | serum | non-lethal | GPX | enzyme | 8 | 0.72 | (Zhang *et al.* 2009) |
| *Gallus gallus* | Aves | natural compound supplementation | serum | non-lethal | SOD | enzyme | 8 | 0.72 | (Zhang *et al.* 2009) |
| *Gallus gallus* | Aves | natural compound supplementation | liver | lethal | MDA | damage | 6 | 0.97 | (Zheng *et al.* 2013) |
| *Gallus gallus* | Aves | natural compound supplementation | liver | lethal | GPX | enzyme | 6 | 0.97 | (Zheng *et al.* 2013) |
| *Gallus gallus* | Aves | natural compound supplementation | liver | lethal | SOD | enzyme | 6 | 0.97 | (Zheng *et al.* 2013) |
| *Gallus gallus* | Aves | natural compound supplementation | liver | lethal | T-AOC | non-enzymatic | 6 | 0.97 | (Zheng *et al.* 2013) |
| *Gallus gallus* | Aves | natural compound supplementation | serum | non-lethal | GPX | enzyme | 6 | 0.97 | (Zheng *et al.* 2013) |
| *Haliotis discus hannai* | Gastropoda | natural compound supplementation | hepatopancreas | lethal | GPX | enzyme | 6 | 2.75 | (Zhang *et al.* 2010) |
| *Haliotis discus hannai* | Gastropoda | natural compound supplementation | hepatopancreas | lethal | GSH | non-enzymatic | 6 | 2.75 | (Zhang *et al.* 2010) |
| *Haliotis discus hannai* | Gastropoda | natural compound supplementation | hepatopancreas | lethal | SOD | enzyme | 6 | 2.75 | (Zhang *et al.* 2010) |
| *Haliotis discus hannai* | Gastropoda | natural compound supplementation | hepatopancreas | lethal | T-AOC | non-enzymatic | 6 | 2.75 | (Zhang *et al.* 2010) |
| *Hirundo rustica* | Aves | antioxidant supplementation | plasma | non-lethal | vitamin E | non-enzymatic | 104 | -0.0001 | (de Ayala, Martinelli & Saino 2006) |
| *Hyphessobrycon callistus* | Actinopterygii | antioxidant supplementation | serum | non-lethal | ALT | damage | 6 | 0.76 | (Wang, Chien & Pan 2006) |
| *Hyphessobrycon callistus* | Actinopterygii | antioxidant supplementation | serum | non-lethal | AST | damage | 6 | 0.76 | (Wang *et al.* 2006) |
| *Hyphessobrycon callistus* | Actinopterygii | antioxidant supplementation | serum | non-lethal | GPX | enzyme | 6 | -0.76 | (Wang *et al.* 2006) |
| *Hyphessobrycon callistus* | Actinopterygii | antioxidant supplementation | serum | non-lethal | SOD | enzyme | 6 | -0.76 | (Wang *et al.* 2006) |
| *Hypophthalmichthys nobilis* | Actinopterygii | stressor exposure | liver | lethal | MDA | damage | 40 | -0.29 | (Sun *et al.* 2013) |
| *Hypophthalmichthys nobilis* | Actinopterygii | stressor exposure | liver | lethal | SOD | enzyme | 40 | 0.29 | (Sun *et al.* 2013) |
| *Ictalurus punctatus* | Actinopterygii | natural compound supplementation | plasma | non-lethal | CAT | enzyme | 6 | 3.49 | (Zheng *et al.* 2009) |
| *Ictalurus punctatus* | Actinopterygii | natural compound supplementation | plasma | non-lethal | SOD | enzyme | 6 | 3.49 | (Zheng *et al.* 2009) |
| *Larus michahellis* | Aves | antioxidant supplementation | RBCs | non-lethal | DNA damage | damage | 70 | -0.09 | (Noguera *et al.* 2011) |
| *Leporinus elongatus* | Actinopterygii | stressor exposure | blood | non-lethal | tGSH | non-enzymatic | 12 | -1.41 | (Filho *et al.* 2005) |
| *Leporinus elongatus* | Actinopterygii | stressor exposure | liver | lethal | tGSH | non-enzymatic | 12 | -1.41 | (Filho *et al.* 2005) |
| *Leporinus elongatus* | Actinopterygii | stressor exposure | blood | non-lethal | GST | enzyme | 12 | 1.41 | (Filho *et al.* 2005) |
| *Litopenaeus vannamei* | Malacostraca | natural compound supplementation | liver | lethal | MDA | damage | 6 | 5.82 | (Shen *et al.* 2010) |
| *Litopenaeus vannamei* | Malacostraca | natural compound supplementation | liver | lethal | GPX | enzyme | 6 | 5.82 | (Shen *et al.* 2010) |
| *Litopenaeus vannamei* | Malacostraca | natural compound supplementation | liver | lethal | T-AOC | non-enzymatic | 6 | 5.82 | (Shen *et al.* 2010) |
| *Litopenaeus vannamei* | Malacostraca | natural compound supplementation | haemolymph | non-lethal | GPX | enzyme | 6 | 3.3 | (Yang *et al.* 2010) |
| *Litopenaeus vannamei* | Malacostraca | antioxidant supplementation | haemolymph | non-lethal | CAT | enzyme | 10 | -4.65 | (Zhang *et al.* 2013b) |
| *Litopenaeus vannamei* | Malacostraca | antioxidant supplementation | haemolymph | non-lethal | SOD | enzyme | 10 | -4.65 | (Zhang *et al.* 2013b) |
| *Litopenaeus vannamei* | Malacostraca | antioxidant supplementation | haemolymph | non-lethal | TAS | non-enzymatic | 10 | 4.65 | (Zhang *et al.* 2013b) |
| *Macrobrachium rosenbergii* | Malacostraca | natural compound supplementation | hepatopancreas | lethal | vitamin C | non-enzymatic | 6 | 7.46 | (Radhakrishnan *et al.* 2013) |
| *Macrobrachium rosenbergii* | Malacostraca | natural compound supplementation | hepatopancreas | lethal | vitamin E | non-enzymatic | 6 | 7.46 | (Radhakrishnan *et al.* 2013) |
| *Macrobrachium rosenbergii* | Malacostraca | natural compound supplementation | muscle | lethal | vitamin C | non-enzymatic | 6 | 7.46 | (Radhakrishnan *et al.* 2013) |
| *Macrobrachium rosenbergii* | Malacostraca | natural compound supplementation | muscle | lethal | vitamin E | non-enzymatic | 6 | 7.46 | (Radhakrishnan *et al.* 2013) |
| *Marsupenaeus japonicus* | Malacostraca | antioxidant supplementation | flesh | lethal | carotenoids | non-enzymatic | 6 | 0.71 | (Chien & Shiau 2005) |
| *Micropterus salmoides* | Actinopterygii | stressor exposure | liver | lethal | CAT | enzyme | 6 | -1.82 | (Chen *et al.* 2013) |
| *Micropterus salmoides* | Actinopterygii | stressor exposure | liver | lethal | MDA | damage | 6 | 1.82 | (Chen *et al.* 2013) |
| *Micropterus salmoides* | Actinopterygii | stressor exposure | muscle | lethal | MDA | damage | 6 | 1.82 | (Chen *et al.* 2013) |
| *Micropterus salmoides* | Actinopterygii | stressor exposure | serum | non-lethal | MDA | damage | 6 | 1.82 | (Chen *et al.* 2013) |
| *Micropterus salmoides* | Actinopterygii | stressor exposure | liver | lethal | GSH | non-enzymatic | 6 | 1.82 | (Chen *et al.* 2013) |
| *Micropterus salmoides* | Actinopterygii | stressor exposure | liver | lethal | vitamin E | non-enzymatic | 6 | -2.29 | (Chen *et al.* 2012) |
| *Micropterus salmoides* | Actinopterygii | stressor exposure | muscle | lethal | vitamin E | non-enzymatic | 6 | -2.29 | (Chen *et al.* 2012) |
| *Micropterus salmoides* | Actinopterygii | stressor exposure | serum | non-lethal | vitamin E | non-enzymatic | 6 | -2.29 | (Chen *et al.* 2012) |
| *Micropterus salmoides* | Actinopterygii | stressor exposure | muscle | lethal | MDA | damage | 6 | -2.29 | (Chen *et al.* 2012) |
| *Micropterus salmoides* | Actinopterygii | stressor exposure | serum | non-lethal | MDA | damage | 6 | -2.29 | (Chen *et al.* 2012) |
| *Oncorhynchus kisutch* | Actinopterygii | antioxidant supplementation | liver | lethal | vitamin E | non-enzymatic | 6 | -0.3 | (Huang *et al.* 2004) |
| *Oncorhynchus kisutch* | Actinopterygii | antioxidant supplementation | muscle | lethal | vitamin E | non-enzymatic | 6 | -0.3 | (Huang *et al.* 2004) |
| *Oncorhynchus kisutch* | Actinopterygii | antioxidant supplementation | liver | lethal | MDA | damage | 6 | -0.3 | (Huang *et al.* 2004) |
| *Oncorhynchus kisutch* | Actinopterygii | antioxidant supplementation | muscle | lethal | MDA | damage | 6 | -0.3 | (Huang *et al.* 2004) |
| *Oncorhynchus mykiss* | Actinopterygii | stressor exposure | whole body | lethal | lipid-soluble fluorescent products | damage | 6 | 0.4 | (Fontagné *et al.* 2008) |
| *Oncorhynchus mykiss* | Actinopterygii | antioxidant supplementation | liver | lethal | GR | enzyme | 12 | -2.35 | (Hisar *et al.* 2012) |
| *Oncorhynchus mykiss* | Actinopterygii | antioxidant supplementation | liver | lethal | MDA | damage | 12 | 2.35 | (Hisar *et al.* 2012) |
| *Oncorhynchus mykiss* | Actinopterygii | antioxidant supplementation | liver | lethal | GST | enzyme | 12 | 2.35 | (Hisar *et al.* 2012) |
| *Oncorhynchus mykiss* | Actinopterygii | antioxidant supplementation | liver | lethal | SOD | enzyme | 12 | 2.35 | (Hisar *et al.* 2012) |
| *Oncorhynchus mykiss* | Actinopterygii | stressor exposure | kidney | lethal | MDA | damage | 6 | 15.91 | (Keleştemur & Seven 2013) |
| *Oncorhynchus mykiss* | Actinopterygii | stressor exposure | liver | lethal | MDA | damage | 6 | 15.91 | (Keleştemur & Seven 2013) |
| *Oncorhynchus mykiss* | Actinopterygii | stressor exposure | muscle | lethal | MDA | damage | 6 | 15.91 | (Keleştemur & Seven 2013) |
| *Oncorhynchus mykiss* | Actinopterygii | natural compound supplementation | kidney | lethal | MDA | damage | 6 | 4.18 | (Keleştemur & Seven 2013) |
| *Oncorhynchus mykiss* | Actinopterygii | natural compound supplementation | liver | lethal | MDA | damage | 6 | 4.18 | (Keleştemur & Seven 2013) |
| *Oncorhynchus mykiss* | Actinopterygii | natural compound supplementation | muscle | lethal | MDA | damage | 6 | 4.18 | (Keleştemur & Seven 2013) |
| *Oncorhynchus mykiss* | Actinopterygii | antioxidant supplementation | kidney | lethal | MDA | damage | 6 | 2.76 | (Keleştemur & Seven 2013) |
| *Oncorhynchus mykiss* | Actinopterygii | antioxidant supplementation | liver | lethal | MDA | damage | 6 | 2.76 | (Keleştemur & Seven 2013) |
| *Oncorhynchus mykiss* | Actinopterygii | antioxidant supplementation | muscle | lethal | MDA | damage | 6 | 2.76 | (Keleştemur & Seven 2013) |
| *Oncorhynchus mykiss* | Actinopterygii | antioxidant supplementation | kidney | lethal | vitamin E | non-enzymatic | 6 | 2.76 | (Keleştemur & Seven 2013) |
| *Oncorhynchus mykiss* | Actinopterygii | antioxidant supplementation | liver | lethal | vitamin E | non-enzymatic | 6 | 2.76 | (Keleştemur & Seven 2013) |
| *Oncorhynchus mykiss* | Actinopterygii | antioxidant supplementation | muscle | lethal | vitamin E | non-enzymatic | 6 | 2.76 | (Keleştemur & Seven 2013) |
| *Oncorhynchus mykiss* | Actinopterygii | natural compound supplementation | kidney | lethal | vitamin C | non-enzymatic | 6 | 4.18 | (Keleştemur & Seven 2013) |
| *Oncorhynchus mykiss* | Actinopterygii | natural compound supplementation | liver | lethal | vitamin C | non-enzymatic | 6 | 4.18 | (Keleştemur & Seven 2013) |
| *Oncorhynchus mykiss* | Actinopterygii | natural compound supplementation | muscle | lethal | vitamin C | non-enzymatic | 6 | 4.18 | (Keleştemur & Seven 2013) |
| *Oncorhynchus mykiss* | Actinopterygii | stressor exposure | kidney | lethal | vitamin A | non-enzymatic | 6 | 15.91 | (Keleştemur & Seven 2013) |
| *Oncorhynchus mykiss* | Actinopterygii | stressor exposure | kidney | lethal | vitamin C | non-enzymatic | 6 | 15.91 | (Keleştemur & Seven 2013) |
| *Oncorhynchus mykiss* | Actinopterygii | stressor exposure | liver | lethal | vitamin A | non-enzymatic | 6 | 15.91 | (Keleştemur & Seven 2013) |
| *Oncorhynchus mykiss* | Actinopterygii | stressor exposure | liver | lethal | vitamin C | non-enzymatic | 6 | 15.91 | (Keleştemur & Seven 2013) |
| *Oncorhynchus mykiss* | Actinopterygii | stressor exposure | muscle | lethal | vitamin A | non-enzymatic | 6 | 15.91 | (Keleştemur & Seven 2013) |
| *Oncorhynchus mykiss* | Actinopterygii | stressor exposure | muscle | lethal | vitamin C | non-enzymatic | 6 | 15.91 | (Keleştemur & Seven 2013) |
| *Oreochromis niloticus* | Actinopterygii | natural compound supplementation | liver | lethal | MDA | damage | 12 | 8.4 | (Özlüer-Hunt *et al.* 2011) |
| *Oreochromis niloticus X O. aureus* | Actinopterygii | antioxidant supplementation | liver | lethal | MDA | damage | 6 | 3.72 | (Huang & Huang 2004) |
| *Oreochromis niloticus X O. aureus* | Actinopterygii | antioxidant supplementation | muscle | lethal | MDA | damage | 6 | 3.72 | (Huang & Huang 2004) |
| *Oreochromis niloticus X O. aureus* | Actinopterygii | antioxidant supplementation | liver | lethal | tGSH | non-enzymatic | 6 | 3.72 | (Huang & Huang 2004) |
| *Oryctolagus cuniculus* | Mammalia | natural compound supplementation | plasma | non-lethal | MDA | damage | 30 | 1.44 | (Liu *et al.* 2010) |
| *Pagrus major* | Actinopterygii | stressor exposure | liver | lethal | vitamin E | non-enzymatic | 6 | -0.0001 | (Gao *et al.* 2013b) |
| *Pagrus major* | Actinopterygii | stressor exposure | liver | lethal | TBARS | damage | 6 | -0.0001 | (Gao *et al.* 2013b) |
| *Pagrus major* | Actinopterygii | stressor exposure | liver | lethal | vitamin E | non-enzymatic | 6 | 0.94 | (Gao *et al.* 2012) |
| *Pagrus major* | Actinopterygii | stressor exposure | muscle | lethal | vitamin E | non-enzymatic | 6 | 0.94 | (Gao *et al.* 2012) |
| *Paralichthys olivaceus* | Actinopterygii | stressor exposure | liver | lethal | TBARS | damage | 6 | 0.94 | (Gao *et al.* 2014b) |
| *Paralichthys olivaceus* | Actinopterygii | stressor exposure | liver | lethal | vitamin C | non-enzymatic | 6 | 0.94 | (Gao *et al.* 2014b) |
| *Paralichthys olivaceus* | Actinopterygii | stressor exposure | liver | lethal | vitamin E | non-enzymatic | 6 | 0.94 | (Gao *et al.* 2014b) |
| *Paralichthys olivaceus* | Actinopterygii | antioxidant supplementation | gill | lethal | vitamin C | non-enzymatic | 6 | 2.05 | (Wang, Kim & Bai 2002) |
| *Paralichthys olivaceus* | Actinopterygii | antioxidant supplementation | kidney | lethal | vitamin C | non-enzymatic | 6 | 2.05 | (Wang *et al.* 2002) |
| *Paralichthys olivaceus* | Actinopterygii | antioxidant supplementation | liver | lethal | vitamin C | non-enzymatic | 6 | 2.05 | (Wang *et al.* 2002) |
| *Paralichthys olivaceus* | Actinopterygii | antioxidant supplementation | muscle | lethal | vitamin C | non-enzymatic | 6 | 2.05 | (Wang *et al.* 2002) |
| *Parus major* | Aves | antioxidant supplementation | RBCs | non-lethal | KRL | non-enzymatic | 48 | 0.71 | (Marri & Richner 2014) |
| *Pelobates cultripes* | Amphibia | stressor exposure | whole body | lethal | CAT | enzyme | 40 | 2.06 | (Gomez-Mestre *et al.* 2013) |
| *Pelobates cultripes* | Amphibia | stressor exposure | whole body | lethal | SOD | enzyme | 40 | 2.06 | (Gomez-Mestre *et al.* 2013) |
| *Pelodiscus sinensis* | Reptilia | antioxidant supplementation | liver | lethal | MDA | damage | 30 | 0.84 | (Huang & Lin 2004) |
| *Pelodiscus sinensis* | Reptilia | antioxidant supplementation | liver | lethal | vitamin E | non-enzymatic | 30 | 0.84 | (Huang & Lin 2004) |
| *Pelodiscus sinensis* | Reptilia | antioxidant supplementation | muscle | lethal | vitamin E | non-enzymatic | 30 | 0.84 | (Huang & Lin 2004) |
| *Penaeus monodon* | Malacostraca | antioxidant supplementation | digestive gland | lethal | MDA | damage | 6 | 7.81 | (Niu *et al.* 2013) |
| *Penaeus monodon* | Malacostraca | antioxidant supplementation | digestive gland | lethal | PCs | damage | 6 | 7.81 | (Niu *et al.* 2013) |
| *Penaeus monodon* | Malacostraca | antioxidant supplementation | digestive gland | lethal | SOD | enzyme | 6 | -7.81 | (Niu *et al.* 2013) |
| *Penaeus monodon* | Malacostraca | antioxidant supplementation | digestive gland | lethal | GPX | enzyme | 6 | 7.81 | (Niu *et al.* 2013) |
| *Penaeus monodon* | Malacostraca | antioxidant supplementation | digestive gland | lethal | TAS | non-enzymatic | 6 | 7.81 | (Niu *et al.* 2013) |
| *Penaeus monodon* | Malacostraca | antioxidant supplementation | haemolymph | non-lethal | ALT | damage | 6 | 1.88 | (Niu *et al.* 2012) |
| *Penaeus monodon* | Malacostraca | antioxidant supplementation | haemolymph | non-lethal | AST | damage | 6 | 1.88 | (Niu *et al.* 2012) |
| *Penaeus monodon* | Malacostraca | antioxidant supplementation | haemolymph | non-lethal | SOD | enzyme | 6 | -1.88 | (Niu *et al.* 2012) |
| *Penaeus monodon* | Malacostraca | antioxidant supplementation | haemolymph | non-lethal | TAS | non-enzymatic | 6 | 1.88 | (Niu *et al.* 2012) |
| *Perca flavescens* | Actinopterygii | antioxidant supplementation | plasma | non-lethal | TBARS | damage | 6 | 2.82 | (Lee & Dabrowski 2003) |
| *Perca flavescens* | Actinopterygii | antioxidant supplementation | liver | lethal | vitamin C | non-enzymatic | 6 | 2.82 | (Lee & Dabrowski 2003) |
| *Perca flavescens* | Actinopterygii | antioxidant supplementation | liver | lethal | vitamin E | non-enzymatic | 6 | 2.82 | (Lee & Dabrowski 2003) |
| *Platichthys stellatus* | Actinopterygii | natural compound supplementation | liver | lethal | MDA | damage | 6 | 2.95 | (Wang *et al.* 2014) |
| *Platichthys stellatus* | Actinopterygii | natural compound supplementation | serum | non-lethal | MDA | damage | 6 | 2.95 | (Wang *et al.* 2014) |
| *Platichthys stellatus* | Actinopterygii | natural compound supplementation | liver | lethal | SOD | enzyme | 6 | 2.95 | (Wang *et al.* 2014) |
| *Platichthys stellatus* | Actinopterygii | natural compound supplementation | liver | lethal | T-AOC | non-enzymatic | 6 | 2.95 | (Wang *et al.* 2014) |
| *Platichthys stellatus* | Actinopterygii | natural compound supplementation | serum | non-lethal | CAT | enzyme | 6 | 2.95 | (Wang *et al.* 2014) |
| *Platichthys stellatus* | Actinopterygii | natural compound supplementation | serum | non-lethal | SOD | enzyme | 6 | 2.95 | (Wang *et al.* 2014) |
| *Rachycentron canadum* | Actinopterygii | antioxidant supplementation | liver | lethal | TBARS | damage | 6 | 3.29 | (Zhou *et al.* 2013) |
| *Rachycentron canadum* | Actinopterygii | antioxidant supplementation | liver | lethal | vitamin E | non-enzymatic | 6 | 3.29 | (Zhou *et al.* 2013) |
| *Rachycentron canadum* | Actinopterygii | antioxidant supplementation | plasma | non-lethal | SOD | enzyme | 6 | 3.29 | (Zhou *et al.* 2013) |
| *Rana temporaria* | Amphibia | stressor exposure | whole body | lethal | MDA | damage | 28 | -1.59 | (Salin *et al.* 2012) |
| *Rattus norvegicus (Sprague-Dawley)* | Mammalia | stressor exposure | liver | lethal | MDA | damage | 12 | 4.74 | (Ohtsuka *et al.* 1998) |
| *Rattus norvegicus (Sprague-Dawley)* | Mammalia | antioxidant supplementation | liver | lethal | GST | enzyme | 12 | -0.37 | (Ohtsuka *et al.* 1998) |
| *Rattus norvegicus (Sprague-Dawley)* | Mammalia | antioxidant supplementation | liver | lethal | vitamin E | non-enzymatic | 12 | -0.37 | (Ohtsuka *et al.* 1998) |
| *Salmo coruhensis* | Actinopterygii | antioxidant supplementation | liver | lethal | CAT | enzyme | 18 | -0.16 | (Can *et al.* 2012) |
| *Salmo coruhensis* | Actinopterygii | antioxidant supplementation | liver | lethal | MDA | damage | 18 | 0.16 | (Can *et al.* 2012) |
| *Spondyliosoma cantharus* | Actinopterygii | natural compound supplementation | plasma | non-lethal | MDA | damage | 6 | 9.61 | (Ma *et al.* 2008) |
| *Spondyliosoma cantharus* | Actinopterygii | natural compound supplementation | liver | lethal | CAT | enzyme | 6 | 9.61 | (Ma *et al.* 2008) |
| *Spondyliosoma cantharus* | Actinopterygii | natural compound supplementation | liver | lethal | GPX | enzyme | 6 | 9.61 | (Ma *et al.* 2008) |
| *Spondyliosoma cantharus* | Actinopterygii | natural compound supplementation | liver | lethal | SOD | enzyme | 6 | 9.61 | (Ma *et al.* 2008) |
| *Spondyliosoma cantharus* | Actinopterygii | natural compound supplementation | muscle | lethal | GST | enzyme | 6 | 9.61 | (Ma *et al.* 2008) |
| *Spondyliosoma cantharus* | Actinopterygii | natural compound supplementation | muscle | lethal | SOD | enzyme | 6 | 9.61 | (Ma *et al.* 2008) |
| *Spondyliosoma cantharus* | Actinopterygii | natural compound supplementation | plasma | non-lethal | GPX | enzyme | 6 | 9.61 | (Ma *et al.* 2008) |
| *Spondyliosoma cantharus* | Actinopterygii | natural compound supplementation | plasma | non-lethal | GST | enzyme | 6 | 9.61 | (Ma *et al.* 2008) |
| *Spondyliosoma cantharus* | Actinopterygii | antioxidant supplementation | liver | lethal | MDA | damage | 6 | 15.49 | (Peng *et al.* 2009) |
| *Spondyliosoma cantharus* | Actinopterygii | stressor exposure | liver | lethal | MDA | damage | 6 | 11.98 | (Peng *et al.* 2009) |
| *Sus scrofa domesticus* | Mammalia | natural compound supplementation | serum | non-lethal | MDA | damage | 8 | 2.34 | (Wang *et al.* 2012a) |
| *Sus scrofa domesticus* | Mammalia | natural compound supplementation | serum | non-lethal | GPX | enzyme | 8 | 2.34 | (Wang *et al.* 2012a) |
| *Sus scrofa domesticus* | Mammalia | natural compound supplementation | serum | non-lethal | SOD | enzyme | 8 | 2.34 | (Wang *et al.* 2012a) |
| *Sus scrofa domesticus* | Mammalia | stressor exposure | serum | non-lethal | MDA | damage | 12 | 0.74 | (Wang, Chi & Kim 2012b) |
| *Trachinotus marginatus* | Actinopterygii | natural compound supplementation | muscle | lethal | TBARS | damage | 6 | -8.1 | (Kütter *et al.* 2012) |

Table S2 Summary details for studies included in cost-MA investigating the effects of growth rate on OS.

| **Species** | **Taxonomic Class** | **Experimental Manipulation** | **Biological Matrix** | **Sampling Method** | **Biomarker** | **Biomarker Category** | **N** | **Hedges' g** | **Reference** |
| --- | --- | --- | --- | --- | --- | --- | --- | --- | --- |
| *Aptenodytes patagonicus* | Aves | none | plasma | non-lethal | ROMs | damage | 28 | 1.1 | (Geiger *et al.* 2011) |
| *Aptenodytes patagonicus* | Aves | none | plasma | non-lethal | OXY | non-enzymatic | 28 | 0.14 | (Geiger *et al.* 2011) |
| *Argopecten ventricosus* | Bivalvia | none | gills | lethal | TBARS | damage | 8 | 1.57 | (Guerra *et al.* 2012) |
| *Argopecten ventricosus* | Bivalvia | none | gills | lethal | PCs | damage | 8 | 3.17 | (Guerra *et al.* 2012) |
| *Argopecten ventricosus* | Bivalvia | none | mantle | lethal | TBARS | damage | 8 | 1.89 | (Guerra *et al.* 2012) |
| *Argopecten ventricosus* | Bivalvia | none | mantle | lethal | PCs | damage | 8 | 0.51 | (Guerra *et al.* 2012) |
| *Argopecten ventricosus* | Bivalvia | none | muscle | lethal | TBARS | damage | 8 | 7.15 | (Guerra *et al.* 2012) |
| *Argopecten ventricosus* | Bivalvia | none | muscle | lethal | PCs | damage | 8 | 1.89 | (Guerra *et al.* 2012) |
| *Argopecten ventricosus* | Bivalvia | none | gills | lethal | CAT | enzyme | 8 | -9.95 | (Guerra *et al.* 2012) |
| *Argopecten ventricosus* | Bivalvia | none | gills | lethal | SOD | enzyme | 8 | -2.66 | (Guerra *et al.* 2012) |
| *Argopecten ventricosus* | Bivalvia | none | mantle | lethal | CAT | enzyme | 8 | -9.92 | (Guerra *et al.* 2012) |
| *Argopecten ventricosus* | Bivalvia | none | mantle | lethal | SOD | enzyme | 8 | -2.79 | (Guerra *et al.* 2012) |
| *Argopecten ventricosus* | Bivalvia | none | muscle | lethal | CAT | enzyme | 8 | -3.14 | (Guerra *et al.* 2012) |
| *Argopecten ventricosus* | Bivalvia | none | muscle | lethal | SOD | enzyme | 8 | -1.05 | (Guerra *et al.* 2012) |
| *Carassius auratus gibelio* | Actinopterygii | dietary changes | intenstine | lethal | SOD | enzyme | 6 | -0.05 | (Dong *et al.* 2013) |
| *Carassius auratus gibelio* | Actinopterygii | dietary changes | intenstine | lethal | CAT | enzyme | 6 | -0.46 | (Dong *et al.* 2013) |
| *Carassius auratus gibelio* | Actinopterygii | dietary changes | intenstine | lethal | GPX | enzyme | 6 | 1.73 | (Dong *et al.* 2013) |
| *Carassius auratus gibelio* | Actinopterygii | dietary changes | intenstine | lethal | GR | enzyme | 6 | 0.12 | (Dong *et al.* 2013) |
| *Carassius auratus gibelio* | Actinopterygii | dietary changes | liver | lethal | SOD | enzyme | 6 | -0.77 | (Dong *et al.* 2013) |
| *Carassius auratus gibelio* | Actinopterygii | dietary changes | liver | lethal | CAT | enzyme | 6 | 2.82 | (Dong *et al.* 2013) |
| *Carassius auratus gibelio* | Actinopterygii | dietary changes | liver | lethal | GPX | enzyme | 6 | 0.18 | (Dong *et al.* 2013) |
| *Carassius auratus gibelio* | Actinopterygii | dietary changes | liver | lethal | GR | enzyme | 6 | 0.96 | (Dong *et al.* 2013) |
| *Carassius auratus gibelio* | Actinopterygii | dietary changes | intenstine | lethal | T-AOC | non-enzymatic | 6 | 0.28 | (Dong *et al.* 2013) |
| *Carassius auratus gibelio* | Actinopterygii | dietary changes | intenstine | lethal | GSH | non-enzymatic | 6 | -0.63 | (Dong *et al.* 2013) |
| *Carassius auratus gibelio* | Actinopterygii | dietary changes | liver | lethal | T-AOC | non-enzymatic | 6 | -1.22 | (Dong *et al.* 2013) |
| *Carassius auratus gibelio* | Actinopterygii | dietary changes | liver | lethal | GSH | non-enzymatic | 6 | -0.13 | (Dong *et al.* 2013) |
| *Chelonia mydas* | Reptilia | compensatory growth | liver | lethal | GPX | enzyme | 17 | 1.74 | (Roark *et al.* 2009) |
| *Chelonia mydas* | Reptilia | compensatory growth | liver | lethal | BIOXYTECH | non-enzymatic | 15 | 1.51 | (Roark *et al.* 2009) |
| *Columba livia* | Aves | dietary changes | serum | non-lethal | ROMs | damage | 21 | 1.72 | (Costantini 2010) |
| *Columba livia* | Aves | dietary changes | serum | non-lethal | OXY | non-enzymatic | 21 | -1.46 | (Costantini 2010) |
| *Columba livia* | Aves | dietary changes | serum | non-lethal | Thiols | non-enzymatic | 21 | 0.17 | (Costantini 2010) |
| *Corvus monedula* | Aves | brood manipulation | plasma | non-lethal | ROMs | damage | 46 | 0.67 | (Salomons 2009) |
| *Corvus monedula* | Aves | brood manipulation | plasma | non-lethal | ROMs | damage | 46 | -0.14 | (Salomons 2009) |
| *Corvus monedula* | Aves | brood manipulation | plasma | non-lethal | OXY | non-enzymatic | 46 | -0.61 | (Salomons 2009) |
| *Cuculus canorus* | Aves | none | plasma | non-lethal | ROMs | damage | 20 | 0.58 | (Hargitai *et al.* 2012) |
| *Cuculus canorus* | Aves | none | plasma | non-lethal | FRAP | non-enzymatic | 21 | -0.57 | (Hargitai *et al.* 2012) |
| *Gallus gallus* | Aves | dietary changes | heart | lethal | MDA | damage | 122 | 0.3 | (Nain *et al.* 2008) |
| *Labeo rohita* | Actinopterygii | dietary changes | liver | lethal | SOD | enzyme | 8 | 2.46 | (Yengkokpam *et al.* 2013) |
| *Labeo rohita* | Actinopterygii | dietary changes | liver | lethal | CAT | enzyme | 8 | 3.45 | (Yengkokpam *et al.* 2013) |
| *Larus michahellis* | Aves | compensatory growth | plasma | non-lethal | trolox | non-enzymatic | 70 | -0.17 | (Noguera *et al.* 2011) |
| *Lestes viridis* | Insecta | compensatory growth | whole body | lethal | SOD | enzyme | 45 | -1.96 | (De Block & Stoks 2008) |
| *Lestes viridis* | Insecta | compensatory growth | whole body | lethal | CAT | enzyme | 45 | -2.82 | (De Block & Stoks 2008) |
| *Litopenaeus vannamei* | Malacostraca | dietary changes | haemolymph | non-lethal | SOD | enzyme | 8 | -0.85 | (Xu & Pan 2014) |
| *Litopenaeus vannamei* | Malacostraca | dietary changes | hepatopancreas | lethal | SOD | enzyme | 8 | 0.36 | (Xu & Pan 2014) |
| *Litopenaeus vannamei* | Malacostraca | dietary changes | haemolymph | non-lethal | T-AOC | non-enzymatic | 8 | -1.8 | (Xu & Pan 2014) |
| *Litopenaeus vannamei* | Malacostraca | dietary changes | haemolymph | non-lethal | GSH:GSSG | non-enzymatic | 8 | -0.77 | (Xu & Pan 2014) |
| *Litopenaeus vannamei* | Malacostraca | dietary changes | hepatopancreas | lethal | T-AOC | non-enzymatic | 8 | -2.56 | (Xu & Pan 2014) |
| *Litopenaeus vannamei* | Malacostraca | dietary changes | hepatopancreas | lethal | GSH:GSSG | non-enzymatic | 8 | 0.28 | (Xu & Pan 2014) |
| *Macrobrachium nipponense* | Malacostraca | compensatory growth | haemolymph | non-lethal | ROIs | damage | 6 | 0.8 | (Li *et al.* 2009) |
| *Macrobrachium nipponense* | Malacostraca | compensatory growth | muscle | lethal | SOD | enzyme | 6 | 0.42 | (Li *et al.* 2009) |
| *Macrobrachium nipponense* | Malacostraca | compensatory growth | muscle | lethal | CAT | enzyme | 6 | 1.2 | (Li *et al.* 2009) |
| *Misgurnus anguillicaudatus* | Actinopterygii | dietary changes | whole body | lethal | CAT | enzyme | 6 | 3.63 | (Gao *et al.* 2014a) |
| *Misgurnus anguillicaudatus* | Actinopterygii | dietary changes | whole body | lethal | SOD | enzyme | 6 | -6.12 | (Gao *et al.* 2014a) |
| *Misgurnus anguillicaudatus* | Actinopterygii | dietary changes | whole body | lethal | GPX | enzyme | 6 | 3.1 | (Gao *et al.* 2014a) |
| *Oreochromis niloticus* | Actinopterygii | dietary changes | liver | lethal | MDA | damage | 12 | -5.19 | (Aziza, Awadin & Orma 2013) |
| *Oreochromis niloticus* | Actinopterygii | dietary changes | muscle | lethal | MDA | damage | 12 | 0 | (Aziza *et al.* 2013) |
| *Oreochromis niloticus* | Actinopterygii | dietary changes | liver | lethal | SOD | enzyme | 10 | 2.84 | (Saïdi *et al.* 2010) |
| *Oreochromis niloticus* | Actinopterygii | dietary changes | liver | lethal | CAT | enzyme | 10 | 2.39 | (Saïdi *et al.* 2010) |
| *Oreochromis niloticus* | Actinopterygii | dietary changes | liver | lethal | GST | enzyme | 10 | 2.35 | (Saïdi *et al.* 2010) |
| *Oreochromis niloticus* | Actinopterygii | dietary changes | liver | lethal | GPX | enzyme | 10 | 2.25 | (Saïdi *et al.* 2010) |
| *Ovis aries (Ile de France X INRA 401)* | Mammalia | compensatory growth | muscle | lethal | PCs | damage | 8 | 0.22 | (Savary-Auzeloux *et al.* 2008) |
| *Ovis aries (Ile de France X INRA 401)* | Mammalia | compensatory growth | muscle | lethal | PCs | damage | 8 | 0.26 | (Savary-Auzeloux *et al.* 2008) |
| *Ovis aries (Ile de France X INRA 401)* | Mammalia | compensatory growth | muscle | lethal | PCs | damage | 8 | -0.26 | (Savary-Auzeloux *et al.* 2008) |
| *Ovis aries (Ile de France X INRA 401)* | Mammalia | compensatory growth | muscle | lethal | GPX | enzyme | 8 | -0.66 | (Savary-Auzeloux *et al.* 2008) |
| *Ovis aries (Ile de France X INRA 401)* | Mammalia | compensatory growth | muscle | lethal | SOD | enzyme | 8 | 0.74 | (Savary-Auzeloux *et al.* 2008) |
| *Ovis aries (Ile de France X INRA 401)* | Mammalia | compensatory growth | muscle | lethal | GPX | enzyme | 8 | -0.84 | (Savary-Auzeloux *et al.* 2008) |
| *Ovis aries (Ile de France X INRA 401)* | Mammalia | compensatory growth | muscle | lethal | SOD | enzyme | 8 | -0.11 | (Savary-Auzeloux *et al.* 2008) |
| *Ovis aries (Ile de France X INRA 401)* | Mammalia | compensatory growth | muscle | lethal | GPX | enzyme | 8 | -0.61 | (Savary-Auzeloux *et al.* 2008) |
| *Ovis aries (Ile de France X INRA 401)* | Mammalia | compensatory growthcompensatory growth | muscle | lethal | SOD | enzyme | 8 | -0.89 | (Savary-Auzeloux *et al.* 2008) |
| *Ovis aries (Ile de France X INRA 401)* | Mammalia | compensatory growth | muscle | lethal | TAS | non-enzymatic | 8 | 3.48 | (Savary-Auzeloux *et al.* 2008) |
| *Ovis aries (Ile de France X INRA 401)* | Mammalia | compensatory growth | muscle | lethal | tGSH | non-enzymatic | 8 | 0.98 | (Savary-Auzeloux *et al.* 2008) |
| *Ovis aries (Ile de France X INRA 401)* | Mammalia | compensatory growth | muscle | lethal | carnosine | non-enzymatic | 8 | 0.15 | (Savary-Auzeloux *et al.* 2008) |
| *Ovis aries (Ile de France X INRA 401)* | Mammalia | compensatory growth | muscle | lethal | anserine | non-enzymatic | 8 | 0.13 | (Savary-Auzeloux *et al.* 2008) |
| *Ovis aries (Ile de France X INRA 401)* | Mammalia | compensatory growth | muscle | lethal | TAS | non-enzymatic | 8 | 1.62 | (Savary-Auzeloux *et al.* 2008) |
| *Ovis aries (Ile de France X INRA 401)* | Mammalia | compensatory growth | muscle | lethal | tGSH | non-enzymatic | 8 | 0.1 | (Savary-Auzeloux *et al.* 2008) |
| *Ovis aries (Ile de France X INRA 401)* | Mammalia | compensatory growth | muscle | lethal | carnosine | non-enzymatic | 8 | 0.59 | (Savary-Auzeloux *et al.* 2008) |
| *Ovis aries (Ile de France X INRA 401)* | Mammalia | compensatory growth | muscle | lethal | anserine | non-enzymatic | 8 | 1.02 | (Savary-Auzeloux *et al.* 2008) |
| *Ovis aries (Ile de France X INRA 401)* | Mammalia | compensatory growth | muscle | lethal | TAS | non-enzymatic | 8 | 2.38 | (Savary-Auzeloux *et al.* 2008) |
| *Ovis aries (Ile de France X INRA 401)* | Mammalia | compensatory growth | muscle | lethal | tGSH | non-enzymatic | 8 | 1.39 | (Savary-Auzeloux *et al.* 2008) |
| *Ovis aries (Ile de France X INRA 401)* | Mammalia | compensatory growth | muscle | lethal | carnosine | non-enzymatic | 8 | -0.51 | (Savary-Auzeloux *et al.* 2008) |
| *Ovis aries (Ile de France X INRA 401)* | Mammalia | compensatory growth | muscle | lethal | anserine | non-enzymatic | 8 | -0.36 | (Savary-Auzeloux *et al.* 2008) |
| *Pagrus major* | Actinopterygii | dietary changes | plasma | non-lethal | ROMs | damage | 6 | 20.66 | (Kader *et al.* 2010) |
| *Pagrus major* | Actinopterygii | dietary changes | plasma | non-lethal | BAP | non-enzymatic | 6 | -0.09 | (Kader *et al.* 2010) |
| *Parus major* | Aves | brood manipulation | plasma | non-lethal | BIOXYTECH | non-enzymatic | 19 | 1.04 | (Kilgas *et al.* 2010) |
| *Pelteobagrus vachelli* | Actinopterygii | dietary changes | intenstine | lethal | SOD | enzyme | 6 | -1.62 | (Dong *et al.* 2013) |
| *Pelteobagrus vachelli* | Actinopterygii | dietary changes | intenstine | lethal | CAT | enzyme | 6 | 0.39 | (Dong *et al.* 2013) |
| *Pelteobagrus vachelli* | Actinopterygii | dietary changes | intenstine | lethal | GPX | enzyme | 6 | -0.3 | (Dong *et al.* 2013) |
| *Pelteobagrus vachelli* | Actinopterygii | dietary changes | intenstine | lethal | GR | enzyme | 6 | -1.1 | (Dong *et al.* 2013) |
| *Pelteobagrus vachelli* | Actinopterygii | dietary changes | liver | lethal | SOD | enzyme | 6 | -1.31 | (Dong *et al.* 2013) |
| *Pelteobagrus vachelli* | Actinopterygii | dietary changes | liver | lethal | CAT | enzyme | 6 | -0.49 | (Dong *et al.* 2013) |
| *Pelteobagrus vachelli* | Actinopterygii | dietary changes | liver | lethal | GPX | enzyme | 6 | -2.51 | (Dong *et al.* 2013) |
| *Pelteobagrus vachelli* | Actinopterygii | dietary changes | liver | lethal | GR | enzyme | 6 | -1.2 | (Dong *et al.* 2013) |
| *Pelteobagrus vachelli* | Actinopterygii | dietary changes | intenstine | lethal | T-AOC | non-enzymatic | 6 | -0.12 | (Dong *et al.* 2013) |
| *Pelteobagrus vachelli* | Actinopterygii | dietary changes | intenstine | lethal | GSH | non-enzymatic | 6 | 0 | (Dong *et al.* 2013) |
| *Pelteobagrus vachelli* | Actinopterygii | dietary changes | liver | lethal | T-AOC | non-enzymatic | 6 | -2.43 | (Dong *et al.* 2013) |
| *Pelteobagrus vachelli* | Actinopterygii | dietary changes | liver | lethal | GSH | non-enzymatic | 6 | -4.72 | (Dong *et al.* 2013) |
| *Periparus ater* | Aves | none | RBCs | non-lethal | 8-OHDG | damage | 14 | 1.27 | (Stier *et al.* 2014) |
| *Periparus ater* | Aves | none | plasma | non-lethal | OXY | non-enzymatic | 14 | -0.54 | (Stier *et al.* 2014) |
| *Rattus norvegicus (Fischer 344)* | Mammalia | dietary changes | blood | non-lethal | 5-OHmdU | damage | 20 | 0.87 | (Djuric *et al.* 2009) |
| *Rattus norvegicus (Fischer 344)* | Mammalia | dietary changes | mammary gland | lethal | 5-OHmdU | damage | 20 | 0.62 | (Djuric *et al.* 2009) |
| *Rattus norvegicus (Sprague-Dawley)* | Mammalia | compensatory growth | muscle | lethal | SOD | enzyme | 16 | 1.22 | (Zheng *et al.* 2012) |
| *Rattus norvegicus (Sprague-Dawley)* | Mammalia | compensatory growth | muscle | lethal | CAT | enzyme | 16 | 1.2 | (Zheng *et al.* 2012) |
| *Rattus norvegicus (Sprague-Dawley)* | Mammalia | compensatory growth | muscle | lethal | GPX | enzyme | 16 | 1.08 | (Zheng *et al.* 2012) |
| *Rattus norvegicus (Wistar)* | Mammalia | dietary changes | brain | lethal | PCs | damage | 10 | -0.22 | (Langley-Evans & Sculley 2006) |
| *Rattus norvegicus (Wistar)* | Mammalia | dietary changes | liver | lethal | PCs | damage | 10 | 1.1 | (Langley-Evans & Sculley 2006) |
| *Rattus norvegicus (Wistar)* | Mammalia | compensatory growth | urine | non-lethal | 8-oxo-dG | damage | 22 | 0.03 | (Tarry-Adkins *et al.* 2008) |
| *Rattus norvegicus (Wistar)* | Mammalia | compensatory growth | urine | non-lethal | 8-oxo-dG | damage | 14 | 0.55 | (Tarry-Adkins *et al.* 2008) |
| *Sparus aurata* | Actinopterygii | dietary changes | liver | lethal | GR | enzyme | 14 | 1.88 | (Sitjà-Bobadilla *et al.* 2005) |
| *Sparus aurata* | Actinopterygii | dietary changes | muscle | lethal | GR | enzyme | 14 | 2.02 | (Sitjà-Bobadilla *et al.* 2005) |
| *Sparus aurata* | Actinopterygii | dietary changes | blood | non-lethal | GSH:GSSG | non-enzymatic | 14 | 1.19 | (Sitjà-Bobadilla *et al.* 2005) |
| *Sparus aurata* | Actinopterygii | dietary changes | liver | lethal | GSH:GSSG | non-enzymatic | 14 | 1.34 | (Sitjà-Bobadilla *et al.* 2005) |
| *Sparus aurata* | Actinopterygii | dietary changes | muscle | lethal | GSH:GSSG | non-enzymatic | 14 | 0.2 | (Sitjà-Bobadilla *et al.* 2005) |
| *Synechogobius hasta* | Actinopterygii | dietary changes | liver | lethal | MDA | damage | 6 | 1.57 | (Luo *et al.* 2012) |
| *Synechogobius hasta* | Actinopterygii | dietary changes | liver | lethal | GPX | enzyme | 6 | -0.49 | (Luo *et al.* 2012) |
| *Synechogobius hasta* | Actinopterygii | dietary changes | liver | lethal | SOD | enzyme | 6 | -4.16 | (Luo *et al.* 2012) |
| *Synechogobius hasta* | Actinopterygii | dietary changes | liver | lethal | CAT | enzyme | 6 | -2.15 | (Luo *et al.* 2012) |
| *Taeniopygia guttata* | Aves | brood manipulation | plasma | non-lethal | carotenoids | non-enzymatic | 52 | 1.5 | (Alonso-Álvarez *et al.* 2007) |
| *Taeniopygia guttata* | Aves | brood manipulation | RBCs | non-lethal | KRL | non-enzymatic | 52 | 0.64 | (Alonso-Álvarez *et al.* 2007) |
| *Taeniopygia guttata* | Aves | brood manipulation | blood | non-lethal | 8-oxo-dG | damage | 16 | 0.26 | Sophie Reichert, unpublished |
| *Taeniopygia guttata* | Aves | brood manipulation | plasma | non-lethal | OXY | non-enzymatic | 16 | -1.2 | Sophie Reichert, unpublished |

Table S3 Pairwise comparisons of the treatment effect on growth for the different experimental approaches (cost-MA). Adjusted p values have been calculated using the sequential Bonferroni correction method. The variance explained by the random factors was 0.33 (study) and 0 (taxonomic class).

| Pairwise comparisons | z value | p value | adjusted p value |
| --- | --- | --- | --- |
| none-compensatory growth | 1.78 | 0.08 | 0.45 |
| none-brood manipulation | 2.24 | 0.03 | 0.15 |
| none-dietary changes | 0.10 | 0.92 | 1.00 |
| compensatory growth-brood manipulation | 0.63 | 0.53 | 1.00 |
| compensatory growth-dietary changes | 2.25 | 0.02 | 0.15 |
| brood manipulation-dietary changes | 2.79 | 0.01 | 0.03 |

**Figure Legends**

Figure S1 Funnel plots for constraint-MA (A) and cost-MA (B) showing the change in effect size (Hedges’ g) with the natural log of sample size (N). The X-axes have been centred on the average effect sizes.

Figure S2 Forest plots for constraint-MA (A) and cost-MA (B). These do not test the main hypotheses but provide evidence that treatments were successful in manipulating OS (A) and growth (B). Since the effect on growth in cost-MA (A) depended on the type of manipulation, the plot has been separated accordingly (there was no difference between experimental manipulations for constraint-MA, therefore an overall effect size is given). Mean and 95% confidence interval, CI, are given on the right hand side. When the CI does not include zero, the effect size is significant. Sample sizes given are number of studies (number of effect sizes). ‘None’ indicates observational studies that found natural and significant growth differences between two groups, for example from different populations or habitats. For full details of growth manipulations, see Table 1 in the main paper.
