# Supplementary figures and images for: Meta‐analysis indicates that oxidative stress is both a constraint on and a cost of growth
Source: Ecol Evol. 2016 Mar 21;6(9):2833–42. doi: 10.1002/ece3.2080 (PMC4863009; doi:10.1002/ece3.2080)

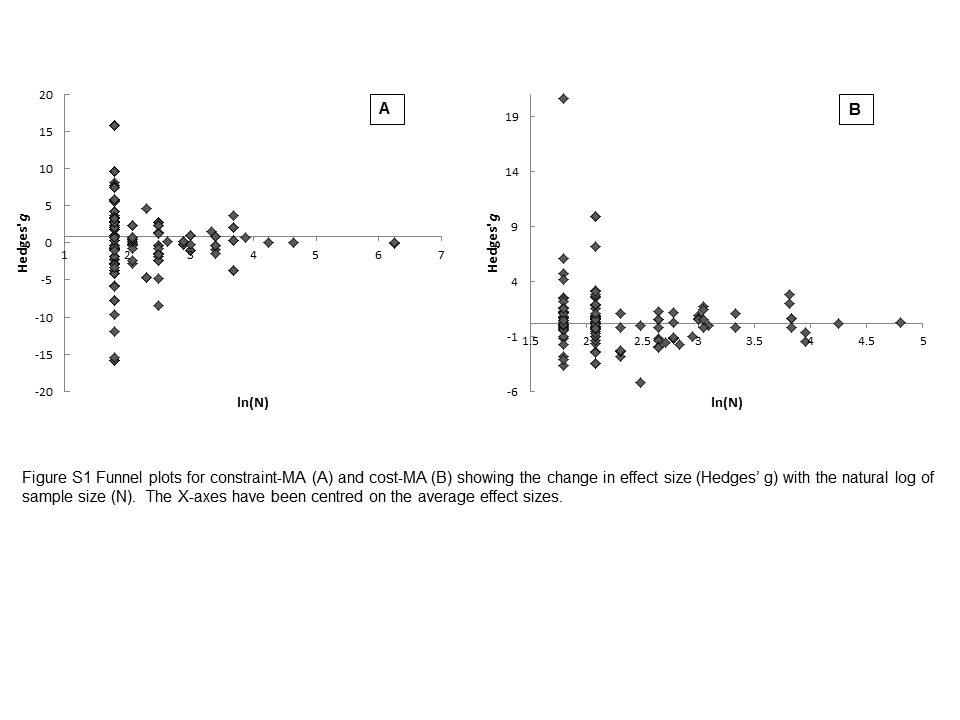

Supplement: Supplementary file 2 [file ECE3-6-2833-s002.tif]

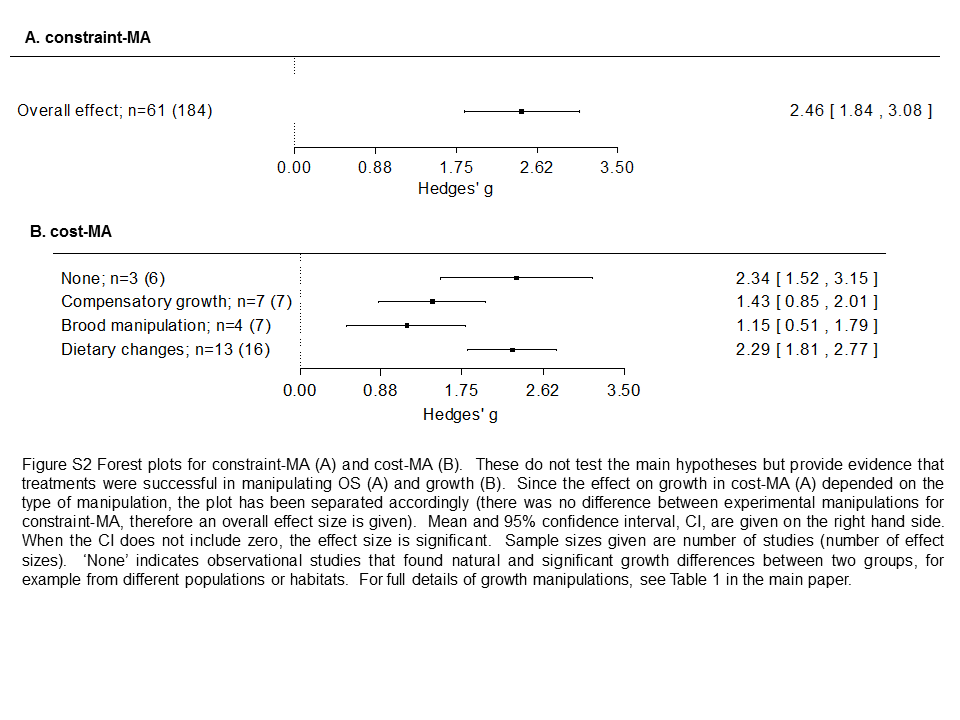

Supplement: Supplementary file 3 [file ECE3-6-2833-s003.tif]
